# A powerful penalized multinomial logistic regression approach

**DOI:** 10.1007/s00180-025-01635-0

**Published:** 2025-05-25

**Authors:** Cornelia Fuetterer, Malte Nalenz, Thomas Augustin, Ruth M. Pfeiffer

**Affiliations:** 1 Institute of AI and Informatics in Medicine, School of Medicine and Health, TUM University Hospital, Technical University of Munich (TUM), Ismaninger Straße 22, 81675 Munich, Germany; 2 Department of Statistics, Ludwig-Maximilians-University Munich, Ludwigstraße 33, 80539 Munich, Germany; 3 Biostatistics Branch, National Cancer Institute, 9609 Medical Center Drive, Bethesda, MD 20892 USA

**Keywords:** Clustering, Penalized regression, Penalty weights, Polytomous logistic regression, Single-cell RNA sequencing data, Shrinkage, Variable selection

## Abstract

Penalized regression methods that shrink model coefficients are popular approaches to improve prediction and for variable selection in high-dimensional settings. We present a penalized (or regularized) regression approach for multinomial logistic models for categorical outcomes with a novel adaptive L1-type penalty term, that incorporates weights based on intra- and inter-outcome category distances of each predictor. A predictor that has large between- and small within-outcome category distances is penalized less and has a higher likelihood to be selected for the final model. We propose and study three measures for weight calculation: an analysis of variance (ANOVA)-based measure and two indices used in clustering approaches. Our novel approach, that we term the *discriminative power lasso* (DP-lasso), thus combines elements of marginal screening with regularized regression methods. We studied the performance of DP-lasso and other published methods in simulations with varying numbers of outcome categories, numbers of predictors, strengths of associations and predictor correlation structures. For correlated predictors, the DP-lasso approach with ANOVA based weights (DPan) resulted in much sparser models than other regularization approaches, especially in high-dimensional settings. When the number *p* of (correlated) predictors was much larger than the available sample size *N*, DPan had the highest true positive rate while maintaining low false positive rates for all simulation settings. Similarly, when ${p<N}$, DPan had high true positive rates and the lowest false positive rates of all methods studied. Thus we recommend DPan for analysing categorical outcomes in relation to high-dimensional predictors. We further illustrate all approaches in ultra high-dimensional settings, using several single-cell RNA-sequencing datasets.

**Supplementary Information:**

The online version contains supplementary material available at 10.1007/s00180-025-01635-0.

## Introduction

Single-cell RNA sequencing (scRNA-seq; see e.g. Tang et al. 2009) allows measuring gene expression for individual cells in a tissue or another type of biological sample. One goal of scRNA-seq analysis is to identify differentially expressed and co-regulated genes in a sample to learn about underlying biological processes. Another goal is to classify the different cells into different known cell types, or sub-populations, based on their genetic information, to aid understanding of the composition of the sample.

In most studies using scRNA-seq, the number *p* of genes is much larger than the number *N* of available samples. A challenge in this and other high-dimensional settings is to identify the often limited number of genes that are truly associated with the given cell types or other characteristics for further studies. A popular approach to identify individual predictors, i.e. components of a vector ${\varvec{X}}=(X_1,\ldots ,X_p$) associated with an outcome *Y* when $p>N$, is penalized (or regularized) regression, such as the lasso (Tibshirani 1996), which selects predictors in a linear regression model by setting coefficients exactly to zero using an L1 norm penalty term. Many other regularization approaches have been proposed, in part to overcome the limitations of the lasso, including its lack of the oracle property, which ensures that the penalized estimator is asymptotically equivalent to the estimator that uses only truly outcome associated variables without penalization. An extension of the lasso that does enjoy the oracle property is the adaptive lasso (Zou 2006), that incorporates predictor-specific weights in the L1 penalty. While the adaptive lasso generally improves variable selection over the standard lasso, it is sensitive to the choice of weights used in the penalty term, computationally complex and does not perform well with highly correlated predictors, particularly when the sample size is small.

Elastic net (Zou and Hastie 2005), another popular penalized likelihood method, extends the lasso by using an L1 and an L2 penalty term and generally performs very well for highly correlated predictors. However, due to the complex interplay between the two penalties elastic net often has difficulty to accurately identify the truly important variables when $p>>N$, and potentially selects irrelevant features. It also can still overfit, and is computationally complex.

To lessen the problem that lasso gives substantially biased estimates for large regression coefficients, Fan and Li (2001) proposed the smoothly clipped absolute deviation (SCAD) penalty which uses a spline-based penalty term. However, in $p>>N$ settings, the computational cost of SCAD optimization can be substantial, and it can become unstable for highly correlated predictors or a very large number of non-zero coefficients. Thus, to effectively apply SCAD in ultra-high dimensional settings, often a pre-processing step like predictor (feature) selection or applying dimension reduction techniques such as principal components analysis, is required to reduce the dimensionality of the predictors. To better accommodate correlated predictors, some approaches proposed for linear models combine likelihood penalization with clustering (Bry et al. 2022; Witten et al. 2014).

The lasso has also been extended to the generalized linear model (GLM) setting, including logistic regression for binary outcomes (Shevade and Keerthi 2003; Roth 2004) and polytomous logistic regression models for categorical outcomes (Krishnapuram et al. 2005). Wang and Wang (2016) derived a weighted elastic-net approach for GLMs for correlated predictors. Wang (2024) recently suggested sparse ridge estimation for highly correlated predictors based on a local linear approximation for GLMs. For polytomous logistic regression models Nibbering and Hastie (2022) suggested a penalization term that weighs the differences of parameters between all combinations of outcome categories.

Here we propose penalized polytomous logistic regression with novel predictor-specific weights in the penalty term, called *discriminatory power (DP)* weights, for regularized regression with categorical outcomes. We present and study three different DP penalty weights based on measures that capture the distances of the predictor components $X_j, j=1,\ldots ,p$ of ${\varvec{X}}$ within and between outcome categories. One weight is based on an analysis of variance (ANOVA)-based measure (Fisher 1992), and two on indices used in clustering approaches, the *Davies-Bouldin index* (Davies and Bouldin 1979), and the *Silhouette index* (Rousseeuw 1987). For all measures a predictor $X_j$ that has small within outcome category distances and large between outcome category distances has a high DP and is penalized less. In contrast, a predictor $X_j$ with large within and small between category distances has a low DP and is penalized more, as it is considered less informative about the outcome *Y*.

The remainder of the paper is structured as follows. In Sect. 2, we give a brief overview of commonly used penalized regression methods and then focus on those for categorical outcomes. Section 3 motivates and introduces the DP penalties for categorical outcomes. In Sect. 4 we assess the performance of our method in simulations, compare it to that of other available approaches, and study the properties of the different weights. In Sect. 5 we illustrate the performance of our DP-lasso and other regularization methods using publicly available scRNA-sequencing datasets. Section 6 concludes with a discussion.

## Background

We briefly review penalized regression methods for generalized linear models and then focus on those for categorical outcomes.

### Overview of penalized regression models

Let *Y* denote the outcome generated from a distribution function *f*, and ${\varvec{X}}^T =(X_{1},\ldots , X_{p})\in \mathbb {R}^p$ a vector of predictors, e.g. gene expression levels, that relates to *Y* via a mean model with link function *h* and parameters $\beta _0$ and ${\varvec{\beta }}^T =(\beta _1,\ldots ,\beta _p)\in \mathbb {R}^p$,

1 $$\begin{aligned} E(Y|{\varvec{X}}) = \mu = h(\eta ) \text{ with } \eta = \beta _0 + \sum _{j=1}^p X_j \beta _j. \end{aligned}$$

When $p>>N$, the parameters ${\varvec{\beta }}$ in (1) are not identifiable. To overcome this issue, penalized regression methods estimate ${\varvec{\beta }}$ using the log-likelihood with an added penalty term to promote sparsity and limit extreme behavior,

2 $$\begin{aligned} \hat{\varvec{\beta }}= \textrm{argmin}_{{\varvec{\beta }}} \Bigl [-\log \{f(Y,{\varvec{\beta }})\}+ \lambda \text {pen}({\varvec{\beta }})\Bigr ]. \end{aligned}$$

The global tuning parameter $\lambda$ regulates the amount of shrinkage of the coefficients ${\varvec{\beta }}$ and is usually chosen via cross-validation. 

Popular penalty terms are the *lasso* (L1; Tibshirani 1996) and *ridge* (L2; Hoerl and Kennard 1970) penalties,

3 $$\begin{aligned} \text {pen}({\varvec{\beta }}) = ||{\varvec{\beta }}||_{1} =\sum _{j=1}^p |\beta _j| \hspace{5pt} \text{ and } \hspace{5pt} \text {pen}({\varvec{\beta }}) = ||{\varvec{\beta }}||^2_{2} = \sum _{j=1}^p \beta _j^2. \end{aligned}$$

The L1 penalty causes coefficients $\beta _j$ to be set to zero, and thus is used for variable selection when *p* is large. However, it can lead to over-shrinkage, i.e. to removing informative predictors. Ridge regression does not set any $\beta$s to zero, and thus yields better predictive performance in the case of multicollinearity in the components of ${\varvec{X}}$  (Hastie et al. 2017).

The *elastic net* penalty combines the L1 and L2 norms into

4 $$\begin{aligned} \text {pen}_\alpha ({\varvec{\beta }}) = (1-\alpha ) \frac{1}{2} ||{\varvec{\beta }}||^2_{2} + \alpha ||{{\varvec{\beta }}}||_{1}, \end{aligned}$$

with $\alpha \in [0,1]$ (Zou and Hastie 2005).

To reduce the amount of over-shrinkage and improve variable selection consistency, Zou (2006) proposed the *adaptive lasso*, that uses predictor-specific penalty weights $w_{j}$,

5 $$\begin{aligned} \hat{\varvec{\beta }}= \textrm{argmin}_{{\varvec{\beta }}} \Bigl [-\log \{f(Y,{\varvec{\beta }})\}+ \lambda \sum _{j=1}^p w_{j}|\beta _{j}| \Bigr ]. \end{aligned}$$

The weights are ${w}_{j}= 1/|\hat{\beta }_{j}|^\gamma$, with $\gamma >0$, where the $\hat{\beta }_{j}$s are estimated from a standard multivariable regression model when $p<N$, or obtained by fitting a penalized likelihood model with a ridge penalty when $p>N$.

A computationally less burdensome extension of the lasso for high-dimensional settings is the *relaxed lasso* (Meinshausen 2007), where model selection and shrinkage estimation are controlled by two separate parameters, $\lambda$ and $\phi \in (0,1]$. When $\phi =1$, the relaxed lasso corresponds to the lasso, but for $\phi <1$ one obtains a solution with coefficients closer to what would be obtained from an orthogonal projection onto the space spanned by the selected variables.

### Penalized regression models for categorical outcomes

We next focus on a special case of (1) for categorical outcomes $Y \in \{1, \ldots , K\}$, the multinomial-logit model with probabilities ${\varvec{\pi }}=(\pi _1,\ldots ,\pi _K)$, where

6 $$\begin{aligned} \begin{aligned} \pi _k({\varvec{x}},{\varvec{\beta }}) = P(Y = k|{\varvec{x}},{\varvec{\beta }})&= \frac{\exp (\eta _k)}{\sum _{r=1}^K \exp (\eta _r)} = \frac{\exp ({\varvec{x}}^T{\varvec{\beta }}_k)}{\sum _{r=1}^K \exp ({\varvec{x}}^T {\varvec{\beta }}_r)}, \hspace{10 pt} k=1,\ldots , K, \end{aligned} \end{aligned}$$

with $\sum _{k=1}^{K} \pi _k=1.$ The category specific parameter vectors ${\varvec{\beta }}_{k}^T=(\beta _{k1}, \ldots , \beta _{kp}),$ $\quad k =1, \ldots , K$, are combined into the $Kp \times 1$ vector ${\varvec{\beta }}^T = (\beta _{11}, \ldots , \beta _{1p}, \ldots , \beta _{K1}, \ldots , \beta _{Kp} )$. Alternatively, one could parameterize (6) using $\tilde{\varvec{\beta }}_k = {\varvec{\beta }}_k-{\varvec{\beta }}_K, k=1,\ldots ,K-1,$ which yields the parameter vector $\tilde{\varvec{\beta }}= (\tilde{\beta }_{11}, \ldots , \tilde{\beta }_{1p}, \ldots , \tilde{\beta }_{(K-1)1}, \ldots , \tilde{\beta }_{(K-1)p} ),$ and the constraint that the $\pi _k$s add up to one is automatically satisfied.

In what follows, we use the $Kp \times 1$ parameter vector ${\varvec{\beta }}$, that is estimated for a sample $(y_i,{\varvec{x}}_i), i=1,\ldots ,N,$ based on the loss function

7 $$\begin{aligned} \hat{\varvec{\beta }}= \textrm{argmin}_{{\varvec{\beta }}} \Bigl [- \frac{1}{N}\sum _{i=1}^N \sum _{k=1}^K {\varvec{1}}(y_i=k) \log \pi _{k} ({\varvec{x}}_i,{\varvec{\beta }}) + \lambda \sum _{k=1}^K \text {pen}_\alpha ({\varvec{\beta }}_k)\Bigr ], \end{aligned}$$

with $\text {pen}_\alpha ({\varvec{\beta }})$ defined in (4) and ${\varvec{1}}(.)$ denoting the indicator function that is one if the argument is true and zero otherwise.

Different choices of $\alpha$ lead to the lasso, ridge or elastic net regularisation for multinomial outcomes. The implementation of the adaptive lasso for multinomial logistic regression in the *R* package *glmnet* (Friedman et al. 2010) uses

8 $$\begin{aligned} \text {pen}({\varvec{\beta }}_k) = \sum _{j=1}^p w_{j}|\beta _{kj}|, \end{aligned}$$

with user-provided variable-specific weights $w_{j}$ in (7).

Nibbering and Hastie (2022) constructed a penalty term using the L2 norm of the difference in group-specific parameters,

9 $$\begin{aligned} pen({\varvec{\beta }}) = \sum _{r >l} w_{rl} ||{\varvec{\beta }}_r - {\varvec{\beta }}_l||_2, \end{aligned}$$

with weights $w_{rl}$ based on class similarities measured by the pairwise distance between class centroids.

Other penalized likelihood models that we use later in our performance comparisons include the *smoothly clipped absolute deviation (SCAD)*, which uses a spline-based penalty term with $\lambda$ and $\tau \lambda$ as knots and hyperparameter $\tau >2$ (Fan and Li 2001; Xie and Huang 2009),

10 $$\begin{aligned} \text {pen(} \beta \text {)} = \lambda | \beta | {\varvec{1}}(| \beta | \le \lambda ) - \frac{\beta ^2 - 2\tau \lambda |\beta |+ \lambda ^2}{2(\tau -1)} {\varvec{1}}( \lambda < |\beta | \le \tau \lambda ) + \frac{\lambda ^2 (\tau +1)}{2} {\varvec{1}}(|\beta | > \tau \lambda ). \end{aligned}$$

We also compare our methods with the *minimax concave penalty (MCP)* (Zhang 2010), with penalty term

11 $$\begin{aligned} \text {pen(}\beta \text {)} = \left(\lambda |\beta | - \frac{\beta ^2}{2\tau }\right) {\varvec{1}}( |\beta | \le \tau \lambda ) + \frac{\tau \lambda ^2}{2} {\varvec{1}}( |\beta | > \tau \lambda ), \end{aligned}$$

with $$\tau >1$$.

## Discriminative power lasso (DP-lasso) for categorical outcomes

Here we propose a novel adaptive version of the lasso for categorical outcomes $Y \in \{1, \ldots , K\}$, the *discriminative power lasso (DP-lasso)*, by introducing weights that use information from the conditional marginal distributions of each $X_j$ given *Y*. For each predictor $X_j$, we define a predictor-specific weight $w^{DP}_{j}$ that aggregates the information in $X_j$ over the groups defined by *Y*, the *discriminative power (DP) weight*. In contrast to weights which are based on coefficients from multivariable regression models, our new weights limit the impact of predictors that only work well in a multivariable model, but are not discriminative univariately. This is particularly important when $p>N$, as overfitting can lead to spurious relationships in multivariable models, and the interplay between different predictors cannot be reliably captured. We thus estimate ${\varvec{\beta }}$ based on the *DP-lasso* loss function

12 $$\begin{aligned} \hat{\varvec{\beta }}^{DP}= \textrm{argmin}_{{\varvec{\beta }}} \Bigl [-\frac{1}{N} \sum _{i=1}^N \sum _{k=1}^K {\varvec{1}}(y_i=k) \log \pi _{k} ({\varvec{x}}_i, {\varvec{\beta }}) + \lambda \sum _{j=1}^p \sum _{k=1}^K {w}^{DP}_{j} |\beta _{kj}| \Bigr ]. \end{aligned}$$

### Measures of discriminative power (DP)

Clustering approaches find groups of observations such that intra-cluster distances, i.e. distances between observations within clusters, are small compared to inter-cluster distances, the distances between clusters. We borrow from these ideas and denote the distance for a component $X_j$ of ${\varvec{X}}$ between observations falling into the same categories defined by *Y*, the *intra-group distance*, by $\delta _j$, and the distance between observations in different *Y* categories (classes), the *inter-group distance*, by $\Delta _j$. However, in contrast to conventional clustering approaches, in our setting the categories defined by *Y* are known. A component $X_j$ has high *discriminative power* if the inter-group distance $\Delta _j$ is large compared to the intra-group distance, $\delta _j$. Such a variable is penalized less, as it is deemed importantly associated with *Y*.

**Fig. 1:**
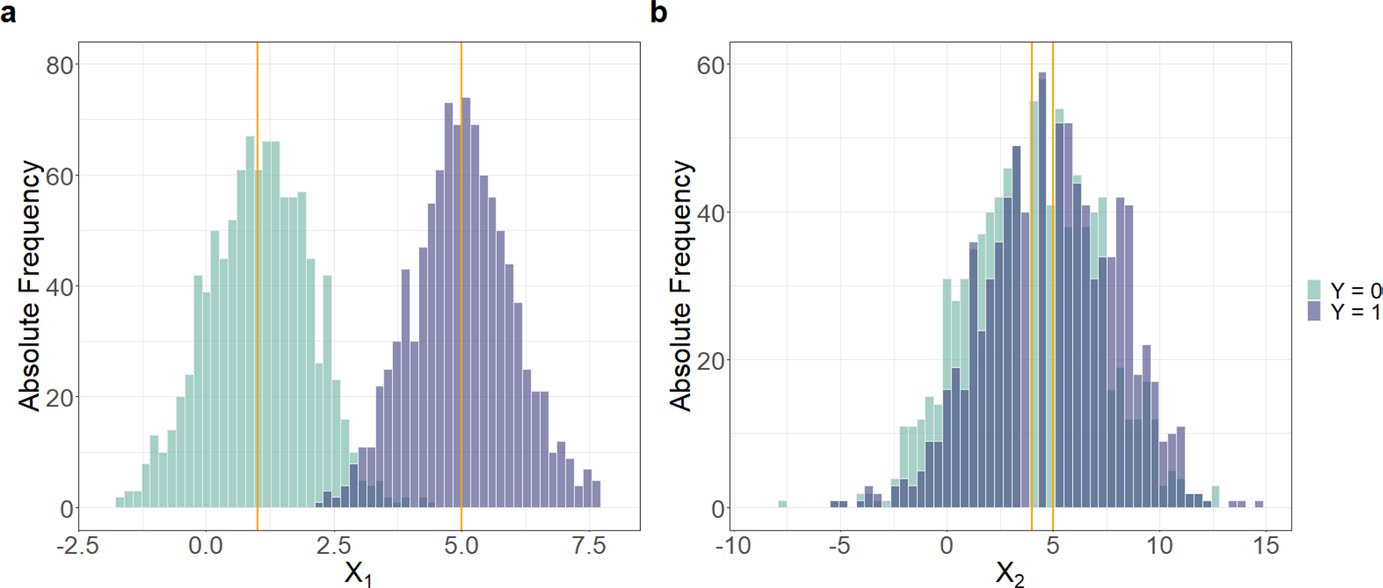
Visualization of the discriminative power (DP): Panels a and b show the histograms of two normally distributed variables $(X_1, X_2)$, in groups $Y=0$ (green) and $Y=1$ (blue): $X_1$ (panel a) has a large $\Delta$, captured by the difference of the means of the *Y* groups ($\mu _{01}-\mu _{11}=4$), and a small $\delta$, measured by the standard deviation ($SD_{01}=SD_{11}=1$); $X_2$ (panel b) shows nearly complete overlap of the distributions in the groups, leading to low discriminative power ($\mu _{02}-\mu _{12}=1$; $SD_{02}=SD_{12}=3$)

This idea is illustrated in Fig. 1 for a binary outcome *Y*. As for $X_1$ the inter-group distance $\Delta _1$, captured by the difference of the means of the *Y* groups ($\mu _{01}-\mu _{11}=4$), is large, and the intra-group distance $\delta _1$ of $X_1$, captured by the within group standard deviations ($SD_{01}=SD_{11}=1$), is small (panel a), $X_1$ has a high DP. In contrast, $X_2$ (panel b) has a low DP, as it has a small inter-group distance $\Delta _2$, and a large intra-group distance $\delta _2$.

We next propose several DP measures to assess the relation of intra- and inter-group distances, based on features of the marginal conditional distribution of *X* given *Y* for a sample $(y_i,{\varvec{x}}_i), i=1, \ldots ,N$. Before calculating the DP measures, we standardize each predictor by subtracting the overall mean and dividing it by its overall standard deviation.

#### Analysis of variance (ANOVA) based measure of DP

ANOVA is a classical way to test whether there is a significant difference between the means of *K* groups based on an F-statistic (Fisher 1992).

Let $\bar{x}_{j}= N^{-1}\sum _{i=1}^{N}x_{ij}$ denote the overall mean of $X_j$ over all *N* observations, and $\bar{x}_{j}^{(k)}=n_k^{-1} \sum _{i=1}^{N}x_{ij} {\varvec{1}}(y_i=k)$ its mean for observations *i* with $y_i=k$, where $n_k=\sum _{i=1}^{N} {\varvec{1}}(y_i=k)$ is the number of observations in class $Y=k$. The corresponding test statistic $F_j$ of $X_j$ is the ratio of the average distances between the groups defined by *Y*, $\tilde{\Delta }_j^{AN}$, and the average distances within the groups, $\tilde{\delta }_j^{AN}$,

13 $$\begin{aligned} F_j = \frac{(N-K)}{(K-1)} \frac{\sum _{k=1}^K n_k(\bar{x}_{j}^{(k)}- \bar{x}_{ j})^2}{\sum _{k=1}^K\sum _{i=1}^{N} {\varvec{1}}(y_i=k)(x_{ij} - \bar{x}_{j}^{(k)})^2} =\frac{\tilde{\Delta }_j^{AN}}{\tilde{\delta }_j^{ AN}}. \end{aligned}$$

$F_j$ is large when $\tilde{\Delta }_j^{AN}$ is larger than $\tilde{\delta }_j^{AN}$.

#### Davies-Bouldin (*DB*) index

The *DB index* was originally developed to assess the quality of clustering based on the compactness and separation of clusters (Davies and Bouldin 1979). The intra-group distances for $X_j$ are defined by the standard deviations of $X_j$ in each *Y* group,

14 $$\begin{aligned} \delta _j^{DB}(k) = \sqrt{ \sum _{i=1}^{N} {\varvec{1}}(y_i=k)(x_{ij} - \bar{x}_{j}^{(k)})^2}, \end{aligned}$$

the inner sum of the denominator in (13). The distance between the groups *r* and *l* is measured by the Euclidean distance of the respective group means $\bar{x}_j^{(r)}$ and $\bar{x}_j^{(l)}$,

15 $$\begin{aligned} \Delta _j^{DB}(r, l) = ||\bar{x}_j^{(r)} - \bar{x}_j^{(l)}||_2. \end{aligned}$$

The overall DB index for $X_j$ is the average over all groups of the maximal ratio of intra- and inter-class distances of two groups,

16 $$\begin{aligned} DB_j =\frac{1}{K} \sum _{k=1}^K \max \limits _{k \ne l}\left\{ \frac{\delta _j^{DB}(k) + \delta _j^{DB}(l)}{\Delta _j^{DB}(k,l)} \right\} . \end{aligned}$$

#### Silhouette (Si) index

The *silhouette index* (Rousseeuw 1987) considers the intra-group and inter-group distances of $X_j$ at the observation level. The intra-group distance of $x_{ij}$ for observation *i* to all observations that are part of the same group as $y_i$ is

17 $$\begin{aligned} \delta _{ij}^{Si}= \frac{1}{(n_k-1)} \sum \limits _{h=1}^{N} {\varvec{1}}(y_i=y_h=k) |x_{ij} - x_{hj}|. \end{aligned}$$

In contrast to $\delta _{ij}^{Si}$, the intra-group distance measures of the ANOVA and DB indices are defined based on the deviations of individual observations from the group mean.

The inter-group distance is based on the minimum average distance of $x_{ij}$ to all the $x_{hj}$ corresponding to $y_h \ne y_i$,

18 $$\begin{aligned} \Delta _{ij}^{Si} = \min \limits _{l \ne y_i}\left\{ \frac{1}{n_l} \sum _{h=1}^{N} |x_{ij}- x_{hj}| I(y_h =l)\right\} . \end{aligned}$$

The silhouette width $s_{ij}$ combines (17) and (18) for observation *i*,

19 $$\begin{aligned} s_{ij} = \frac{\Delta _{ij}^{Si}- \delta _{ij}^{Si}}{\max \{\delta _{ij}^{Si}, \Delta _{ij}^{Si}\}}. \end{aligned}$$

Note that when the intra-group distance $\delta _{ij}^{Si}$ is larger than the inter-group distance $\Delta _{ij}^{Si}$, $s_{ij}$ will be negative, indicating that this observation should be in a different group.

In the last step, the silhouette index $S_j$, which takes values in $[-1,1]$, is calculated by averaging the silhouette widths of all *N* observations in the sample,

20 $$\begin{aligned} S_j =\frac{1}{N} \sum _{i=1}^{N} s_{ij}. \end{aligned}$$

### Discriminative power (DP) weights and DP model fitting

The components of ${\varvec{X}}$ that are informative about the outcome *Y* have high DPs and should be penalized less in (12). Using the DP indices introduced in Sect. 3.1, we define weights for $X_j, j=1,\ldots ,p,$

$$w_{j}^{{DP}} = \left\{ \begin{gathered} w_{j}^{{AN}} = \log \left( {1 + F_{j}^{{ - 1}} } \right)\quad (21) \hfill \\ w_{j}^{{DB}} = DB_{j}\quad \quad \quad \quad \quad  (22) \hfill \\ w_{j}^{{Si}} = \frac{1}{{\left| {S_{j} } \right|}} \quad \quad \quad \quad \quad \quad (23). \hfill \\ \end{gathered} \right.$$

As the ANOVA-based DP measure $F_j$ can become quite large, we use a logarithmic transformation to attenuate the differences in weights between the predictors and to avoid numerical instability.

We refer to the DP-lasso approaches that use the weights in (21)–(23) as *DPan*, *DPdb*, and *DPsi*, respectively. The pseudocode in Algorithm 1 describes the most important steps of the DP-lasso fitting. An *R* program for fitting the DP-lasso is available at https://github.com/montnelia/DP.lasso.

**Algorithm 1:**
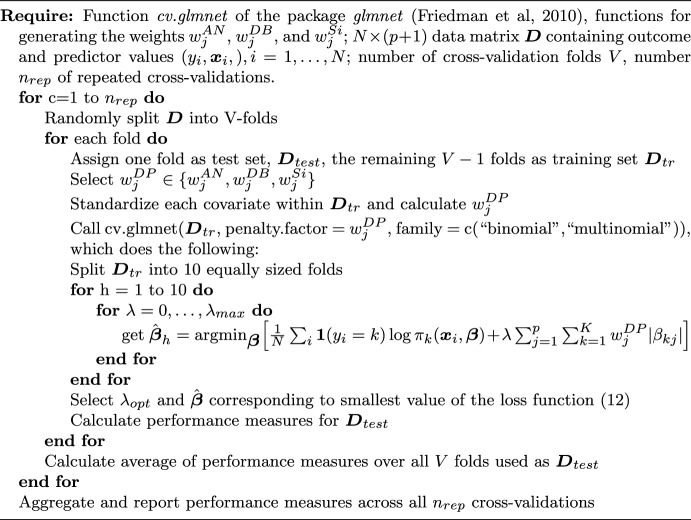
Fitting the DP-lasso in equation (12)

## Simulation studies

We assessed the performance of the DP-lasso for the different choices of weights in various simulated settings and compared it with that of other regularization methods.

### Data generation

First, we drew predictors from a multivariate normal distribution, ${\varvec{x}}_i \sim MVN(0, {\varvec{\Sigma }}),$ $i=1,\ldots ,N$ for various choices of ${\varvec{\Sigma }}$, all of the form

24 $$\begin{aligned} {\varvec{\Sigma }}= \left[ \begin{array}{ccccc} {\varvec{B}}& \quad {\varvec{0}} & \quad \cdots & \quad & \quad {\varvec{0}} \\ {\varvec{0}} & \quad {\varvec{B}}& \quad \cdots & \quad & \quad {\varvec{0}} \\ \vdots & \quad \vdots & \quad & \quad \vdots \\ {\varvec{0}} & \quad \cdots & \quad & \quad & \quad {\varvec{B}}\\ \end{array}\right] . \end{aligned}$$

The matrix ${\varvec{B}}$ in (24) had dimension $20 \times 20$ and entries $[{\varvec{B}}]_{jj}=\sigma ^2=0.5, j=1,\ldots ,20,$ and $[{\varvec{B}}]_{jl}=\rho \sigma ^2, j \ne l$, for $\rho = 0, 0.3, 0.6$ and $\rho = 0.9$. Given ${\varvec{x}}_i$, we next generated $y_i$ from a multinomial distribution with probabilities given in (6). For $K=2$ groups we used ${\varvec{\beta }}_{1}={\varvec{0}}$ and $\beta _{2j}=\kappa , j=1,\ldots ,10$, and $\beta _{2j}=0, j=11,\ldots ,p$. For $K=4$ and $K=7$ groups we studied two choices of parameter values ${\varvec{\beta }}$ in (6).

For the *structure 1* setting with $K=4$, the true parameters were ${\varvec{\beta }}_{1}={\varvec{0}}$, $\beta _{2j}=\kappa , \beta _{3j}=-\kappa , \beta _{4j}=\kappa$ for $j=1, \ldots , 10$ and all other components of ${\varvec{\beta }}_{2}$, ${\varvec{\beta }}_{3}$ and ${\varvec{\beta }}_{4}$ were zero. For $K=7$ groups we added three parameter vectors with $\beta _{5j}=\kappa$, $\beta _{6j}=-\kappa$ and $\beta _{7j}=\kappa$, for $j=1,\ldots ,10$, and all other components were set to zero. We provide simulation results for $\kappa =0.5$ and 0.2.

Under *structure 2* we let $\beta _{3j}=-\kappa ,j=11, \ldots , 20$ for $K=4$ groups, and $\beta _{3j}=-\kappa , \beta _{6j}=-\kappa , j=11, \ldots , 20$ for $K=7$ groups, with all other components of ${\varvec{\beta }}_{3}$ and ${\varvec{\beta }}_{6}$ equal to zero. We studied $p=100, 1000$ and 10,000 predictors, with sample sizes $N=500$ and $N=1000$ for $K=4$ and $N=1000$ for $K=7$.

### Methods and algorithms used in comparison with DP-lasso

We included the following methods in the comparison with our DP-lasso approaches: the minimax concave penalty (MCP), the smoothly clipped absolute deviation (SCAD), the Lasso, the Elastic net (Enet), and the adaptive lasso with two different sets of weights, AdaLS and AdaRI. We used *glmnet* (Friedman et al. 2010) to fit Lasso, Enet, AdaLS and AdaRI and *ncvreg* (Breheny and Huang 2011) to fit MCP and SCAD with the default settings, except for the maximum number of iterations which we increased to 100,000. This made the results more comparable to those of *glmnet*, which uses 100,000 iterations as the default in the optimization. For Enet, $\alpha$ in equation (4) was optimized based on cross-validation applied to the training dataset. The weights for the adaptive lasso approach AdaLS were the averages of the absolute values of the coefficients from the generalized least squares model fit to each predictor separately (using the function *multinom* in the *R* package *nnet* (Venables and Ripley 2002)), and for AdaRI we used weights from a ridge regression fit based on all predictors jointly (*cv.glmnet* with $\alpha =0$). Of note, the available implementations of MCP and SCAD only allow analysing binary outcome data, and thus these methods are not included in comparisons for more than $K=2$ outcome groups.

The following *R* functions and packages were used in the DP weight computation: *aov* (*R* package *stats*), *index.DB* (*R* package *clusterSim*; Walesiak and Dudek (2020)) and *silhouette* (*R* package *cluster*; Maechler et al. 2022). Both the DP weights and the weights for the adaptive lasso were passed with the penalty.factor argument in *glmnet*. As a note, within *glmnet* any penalty weights are rescaled so that they sum up to *p*.

Each method was evaluated based on cross-validation, repeated on 100 random splits of each dataset.

**Table 1:** Algorithms and R packages used for the analysis and the simulation studies

Method	Algorithm/R package
DP-lasso with ANOVA weights (DPan)	*glmnet* (version 4.1.1), *stats* (version 4.2.2)
DP-lasso with DB weights (DPdb)	*glmnet* (version 4.1.1), *clusterSim* (version 0.51.3)
DP-lasso with Si weights (DPsi)	*glmnet* (version 4.1.1), *cluster* (version 2.1.4)
MCP	*ncvreg* (version 3.13.0)
SCAD	*ncvreg* (version 3.13.0)
Lasso	*glmnet* (version 4.1.1)
Elastic net (Enet)	*glmnet* (version 4.1.1)
Adaptive lasso with Ridge penalties (AdaRI)	*glmnet* (version 4.1.1)
Adaptive lasso with least squares penalty (AdaLS)	*glmnet* (version 4.1.1), *nnet* (version 7.3.19)

Table 1 gives further details for the *R* packages used for all the methods in our simulation study.

We could not evaluate some of the other penalized approaches for categorical outcomes due to lack of available *R* software. The *R* package *gvcm.cat* (Oelker 2013) that implements the approach by Gertheiss and Tutz (2010) is no longer available on CRAN, and the multiclass-penalized regression with weighted penalty parameters by Nibbering and Hastie (2022) is not implemented in *R*.

### Performance measures

We assessed the performance of the different methods by the *true positive rate (TPR) * and the *false positive rate (FPR)*. The TPR is the proportion of predictors correctly identified by the model as being associated with the outcome,

25 $$\begin{aligned} \text {TPR} = \frac{\sum _{j =1}^{p} {\varvec{1}}( \sum _{k =1}^{K} |\hat{\beta }_{kj}| \ne 0) {\varvec{1}}( \sum _{k =1} |\beta _{kj} | \ne 0)}{ \sum _{j =1}^{p} {\varvec{1}}( \sum _{k =1}^K |\beta _{kj} | \ne 0)}. \end{aligned}$$

The FPR is the proportion of predictors incorrectly detected as associated with the outcome, out of all predictors that are not outcome associated,

26 $$\begin{aligned} \text {FPR} = \frac{\sum _{j =1}^{p} {\varvec{1}}( \sum _{k =1}^{K} |\hat{\beta }_{kj}| \ne 0) {\varvec{1}}( \sum _{k =1} |\beta _{kj} | = 0)}{ \sum _{j =1}^{p} {\varvec{1}}( \sum _{k =1}^K |\beta _{kj} | = 0)}. \end{aligned}$$

The total number of selected predictors is calculated as

27 $$\begin{aligned} \# \text {predictors } = \sum _{j =1}^{p} {\varvec{1}}( \sum _{k =1}^{K} |\hat{\beta }_{kj}| \ne 0). \end{aligned}$$

### Simulation results

We summarize the results for the various methods for data with $K=7$ outcome groups generated from structure 1 with $\kappa =0.5$ for $N=1000$ samples with $p=100, 1000$, and 10,000 predictors in the main paper. Results for data generated from structures 1 and 2 with $\kappa =0.5$ and $\kappa =0.2$, and for $K=4$ for $N=1000$ and $N=500$ and for $K=2$ and $N=500$ are presented in Supplemental Tables 1 to 15.

**Fig. 2:**
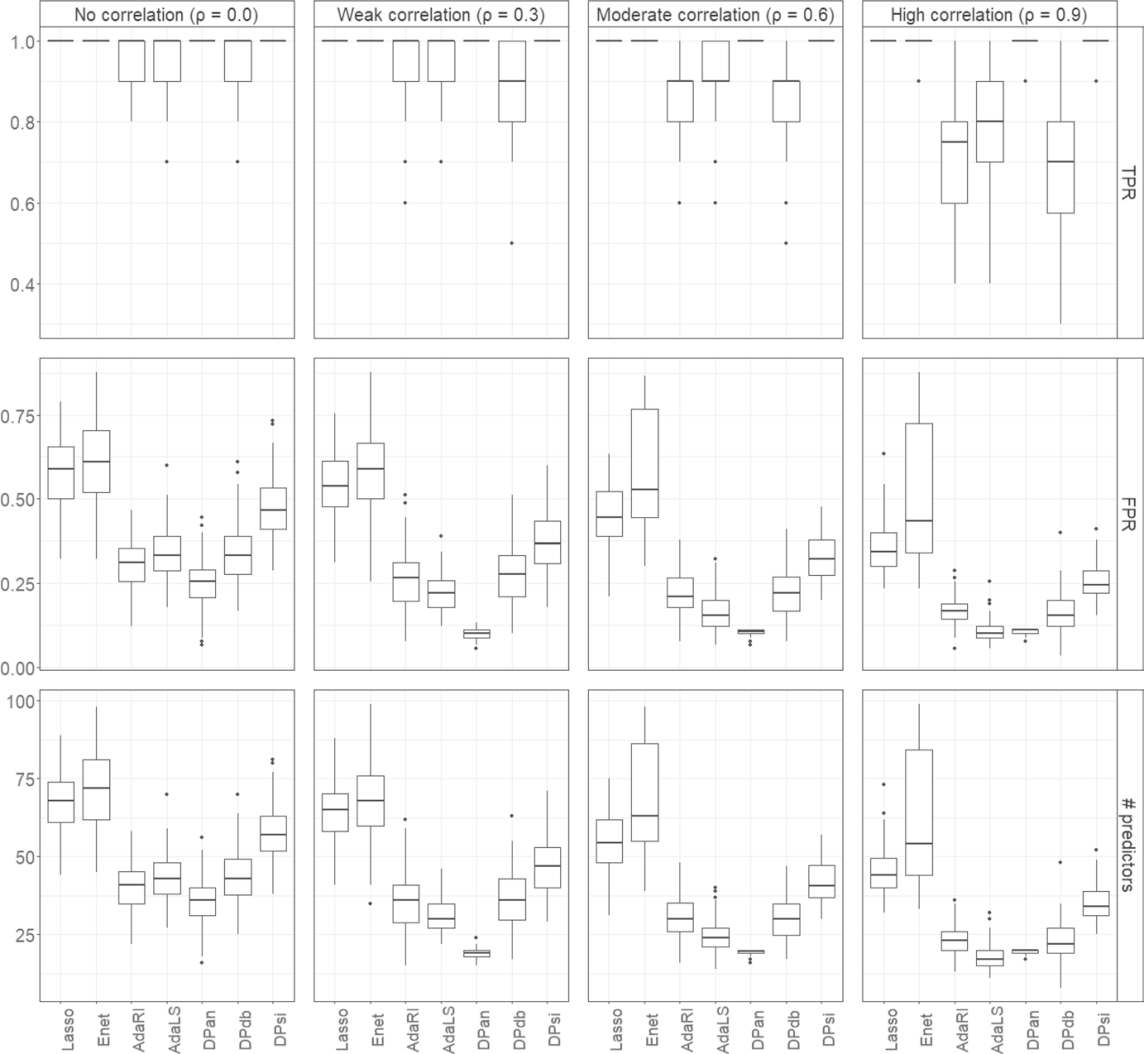
True positive rates (TPRs) and false positive rates (FPRs) for $K=7$ outcome categories, $p=100$, and $N=1000$ for different correlations ρ. Data were generated from scenario 1, i.e. in (6) we let $\beta _{2j}=\kappa , \beta _{3j}=-\kappa , \beta _{4j}=\kappa , \beta _{5j}=\kappa , \beta _{6j}=-\kappa$ and $\beta _{7j}=\kappa$, for $j=1,\ldots ,10,$ with $\kappa =0.5$ and all other components $\beta _{kj}=0$. The results are averaged over 100 runs

#### Results for variable selection

For $p=100$ with $N=1000$ and $\rho =0$, all methods had TPRs close to one, i.e. correctly identified the 10 outcome associated predictors (Fig. 2, Supplemental Table 1). As $\rho$ increased, the TPRs of DPan, DPsi, Lasso and Enet remained one, but the TPRs of AdaRI, AdaLS and DPdb were close to or below 80%. For all values of $\rho$, DPan had the lowest FPR, while the FPRs of Lasso and Enet ranged from 40% to 60%. A similar pattern was seen for the number of selected predictors. DPan selected the lowest number of predictors of all methods for all values of $\rho$. For uncorrelated predictors, the average number was 40, which dropped to below 20 for $\rho \ne 0$ (Table 2). Again, Lasso and Enet selected the largest number of predictors of all methods. For $\rho =0.9$, the FPR and number of predictors for AdaRI and AdaLS were similar to those for DPan.

**Fig. 3:**
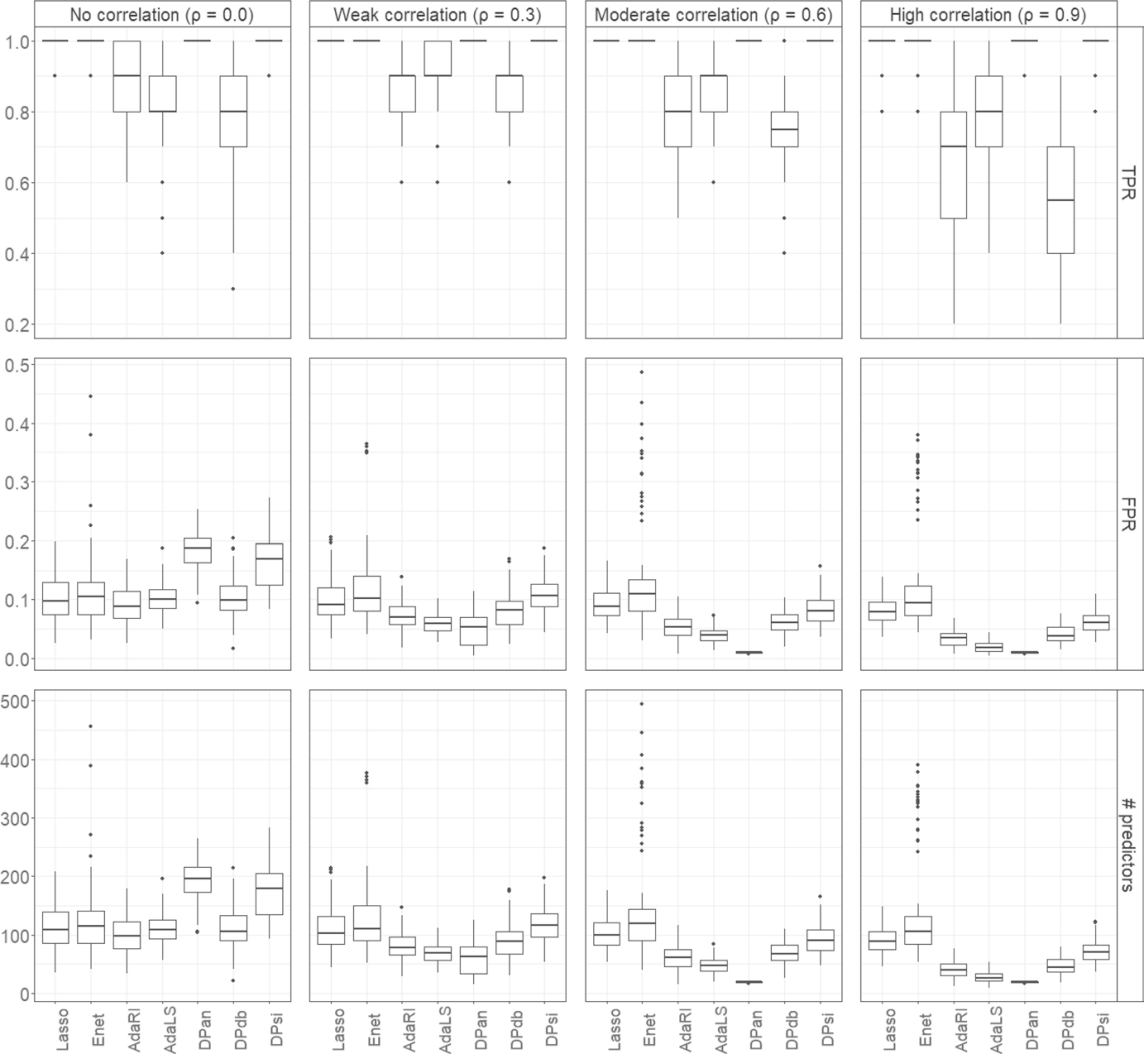
True positive rates (TPRs) and false positive rates (FPRs) for $K=7$ outcome categories, $p=N=1000$ for different correlations ρ. Data were generated from scenario 1, i.e. in (6) we let $\beta _{2j}=\kappa , \beta _{3j}=-\kappa , \beta _{4j}=\kappa , \beta _{5j}=\kappa , \beta _{6j}=-\kappa$ and $\beta _{7j}=\kappa$, for $j=1,\ldots ,10,$ with $\kappa =0.5$ and all other components $\beta _{kj}=0$. The results are averaged over 100 runs

The results for structure 1 with lower signal strength, $\kappa =0.2$, and for structure 2 were similar (Supplemental Table 1).

Figure 3 and Supplemental Tables 3 and 4 show the results when $p=N= 1000$. Lasso, Enet, DPan and DPsi had TPRs close to one for all values of $\rho$. The TPRs of AdaRI, AdaLS and DPdb decreased with increasing $\rho$ and were below 80% for $\rho =0.9$ for all three methods. For $\rho =0$, the FPRs of DPan and DPsi were around 20% and around 10% for all other methods. However, as $\rho$ increased, the FPR for DPan dropped to nearly zero and was the lowest of all methods. A similar pattern was seen for the number of selected predictors, which was highest for DPan for $\rho =0$ (around 200), but much lower, close to 20, for the highest correlation. Interestingly, Enet had highly variable FPRs and numbers of selected predictors, up to 500 for $\rho =0.6$.

**Fig. 4:**
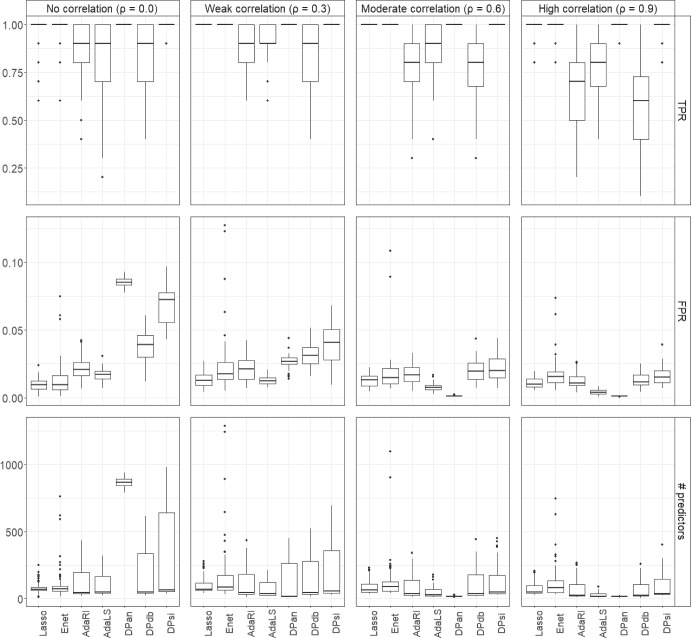
True positive rates (TPRs) and false positive rates (FPRs) for $K=7$ outcome categories, $p=10{,}000$ and $N=1000$ for different correlations ρ. Data were generated from scenario 1, i.e. in (6) we let $\beta _{2j}=\kappa , \beta _{3j}=-\kappa , \beta _{4j}=\kappa$, $\beta _{5j}=\kappa$, $\beta _{6j}=-\kappa$ and $\beta _{7j}=\kappa$, for $j=1,\ldots ,10,$ with $\kappa =0.5$ and all other components $\beta _{kj}=0$. The results are averaged over 100 runs

For $p=10{,}000$ predictors and $N=1000$ the patterns were generally similar to the other *p* and *N* settings, but somewhat more pronounced (Fig. 4). For $\rho =0$ the DP methods had the highest FPRs, but all below 10%, and Lasso and Enet had the lowest FPRs, and DPan selected the largest number of predictors (around 900). However, for high correlations ($\rho =0.6$ and 0.9), DPan had FPRs close to zero and also selected the lowest number of predictors of all methods (Supplemental Tables 5 and Table 6).

Results were qualitatively similar when data were generated under model structure 2, or with $K=2$ or $K=4$ classes for varying number of predictors *p* and samples sizes *N*. For all settings with $K=2$, $K=4$ and $K=7$, when the associations were weaker ($\kappa =0.2$), the patterns of performance across methods were similar to those for $\kappa =0.5$, and FPRs remained low, but TPRs were lower than for $\kappa =0.5$ (see Supplemental Tables 7 to 15).

#### Comparison of the penalty weights

To gain further insight into the performance of the different methods, we compared the weights used in the penalty functions of DP-lasso and the adaptive lasso procedures.

**Fig. 5:**
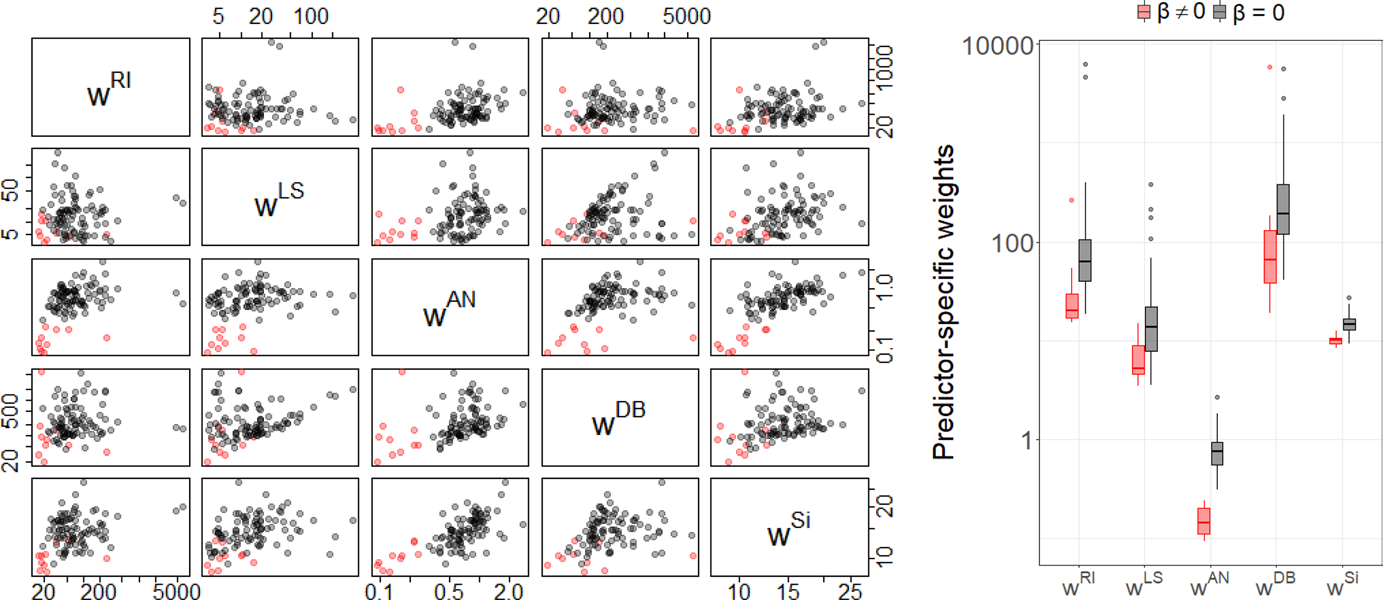
Weights for data generated from scenario 1 with $\rho =0$, $\kappa =0.5$, $p=100$, $N=1000$ for outcomes with $K=7$ categories. Red dots/boxes correspond to variables that were simulated as outcome associated. All axes are on the logarithmic scale

**Fig. 6:**
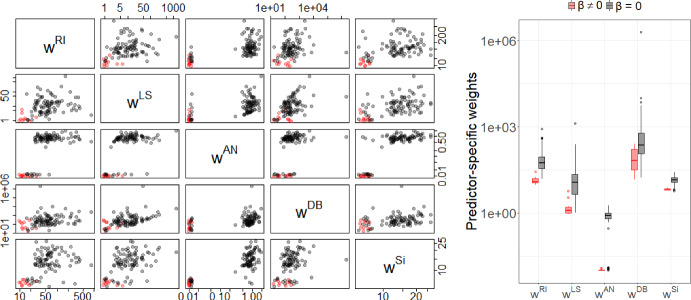
Weights for data generated from scenario 1  with $\rho =0.6$, $\kappa =0.5$, $p=100$, $N=1000$ for outcomes with $K=7$ categories. Red dots/boxes correspond to variables that were simulated as outcome associated. All axes are on the logarithmic scale

Figures 5 and 6 show pairwise scatter plots and box plots of all predictor-specific weights for $p=100$, $N=1000$, for data generated from scenario 1 with $K=7$ outcome groups for $\rho =0$ and for $\rho =0.6$, respectively. Red dots/boxes correspond to weights for the 10 predictors that were associated with the outcome, and the black dots/boxes correspond to the weights for unassociated predictors. For all methods the weights for outcome associated predictors were lower than for “null” predictors, i.e. the outcome associated predictors were penalized less. For all methods the separation of the weights for associated and non-associated predictors was more pronounced for $\rho =0.6$ than for $\rho =0$ as can be seen in the box plots in Figs. 5 and 6. The best separation of weights for associated and null predictors for both correlations, $\rho =0$ and $\rho =0.6$, was seen for the ANOVA based weights, which explains the superior performance of DPan in the variable selection.

Plots for predictor-specific weights for $p=100$ and $p=10{,}000$ for $K=7$ for different values of $\rho$ are given in Supplemental Figures 1-6.

## Analyses of single-cell RNA-sequencing data

To further illustrate the methods, we analyzed four single-cell RNA-sequencing datasets, summarized in Table 2. As proposed by Soneson and Robinson (2018), we only included genes with read counts larger than 1 transcript per million mapped reads (TPM) in at least 25$\%$ of the considered cells in our analysis. Supplemental Table 16 lists the websites where the datasets are publicly available for download.

The dataset EMTAB2805 (Buettner et al. 2015) contains single-cell RNA-seq data from mouse embryonic stem cells (mESCs) at different cell cycle phases. A total of 288 single cells were investigated, 96 cells in each of the cell cycle stages *G*1, *G*2*M* and *S*. For the categorical cell cycle stage outcome with $K=3$, $p=12849$ predictors were included in the model.

The dataset GSE45719 (Deng et al. 2014) contains mouse strain crosses of the first generation, measuring the monoallelic expression among different stages.

**Table 2:** Number of predictors *p* and number of observations $N_i, i=1,\ldots ,K$ in each of the  $K=3$ or $K=4$ outcome categories for four single-cell RNA-sequencing datasets

Dataset	EMTAB2805	GSE45719	GSE48968	GSE74596
	$p=12849$	$p=10851$	$p=7831$	$p=7329$
	K=3	K=3	K=3	K=4
Group 1	*G1*	*mid blast*	*LPS 1 h*	*NKT0*
$N_1$	96	60	96	45
Group 2	*G2M*	*16-cell*	*LPS 4 h*	*NKT17*
$N_2$	96	50	191	44
Group 3	*S*	*8-cell*	*LPS 6 h*	*NKT1*
$N_3$	96	37	191	46
Group 4	–	–	–	*NKT2*
$N_4$	–	–	–	68

We included the following numbers of cells: 60 mid blastocyst *(mid blast)*, 50 16-cell embryos *(16-cell)*, and 37 8-cell embryos *(8-cell)* cells. Thus there are $K=3$ outcome categories, and we used $p=10851$ expression levels in the models.

The GSE48968 data (Shalek et al. 2014) arose from dendritic cells from the bone-marrow of one mouse that was stimulated with Lipopolysaccharides (LPS) for different duration times. To assess the relationship between duration time and expression as a measure of immune response, we analyzed 96 cells stimulated for 1 h (*LPS 1 h*), 191 cells stimulated for 4 h (*LPS 4 h*), and 191 cells stimulated for 6 h (*LPS 6 h*). Thus, three different stimulation times ($K=3$) and $p=7831$ genes were included in the analysis.

The dataset GSE74596 (Engel et al. 2016) contains different types of natural killer T (NKT) cells that regulate the immune response, extracted from the thymus of a mouse. The cell types examined were *NKT0* cells ($N=45$), *NKT17* ($N=44$), *NKT1* cells ($N=46$), and *NKT2* cells ($N=68$). Thus, we studied an outcome with $K=4$ categories and $p=7329$ genes.

**Table 3:** Number of selected genes and the misclassification rates with corresponding standard deviations (SD) in parentheses for the four single-cell RNA-sequencing datasets estimated based on cross validation

	EMTAB2805	GSE45719	GSE48968	GSE74596
	$K=3$	$K=3$	$K=3$	$K=4$
*Mean number of selected variables (SD)*				
Lasso	132 (0.08)	68 (0.02)	168 (0.51)	72 (0.14)
Enet	636 (20.78)	731 (5.24)	829 (10.82)	441 (20.48)
AdaRI	72 (1.38)	35 (1.19)	100 (2.02)	61 (2.37)
AdaLS	47 (3.21)	6 (0.25)	5 (0.21)	38 (1.60)
DPan	44 (0.17)	24 (0.01)	70 (0.12)	17 (0.01)
DPdb	72 (0.00)	38 (0.04)	126 (0.17)	37 (0.17)
DPsi	184 (0.07)	33 (0.10)	176 (0.48)	88 (0.26)
*Misclassification rate (SD)*				
Lasso	0.05 (0.001)	0.03 (0.000)	0.17 (0.001)	0.01 (0.001)
Enet	0.06 (0.002)	0.03 (0.001)	0.17 (0.003)	0.01 (0.000)
AdaRI	0.00 (0.011)	0.13 (0.018)	0.27 (0.012)	0.30 (0.022)
AdaLS	0.00 (0.013)	0.28 (0.009)	0.32 (0.007)	0.16 (0.018)
DPan	0.06 (0.000)	0.08 (0.005)	0.17 (0.001)	0.02 (0.001)
DPdb	0.10 (0.001)	0.06 (0.001)	0.18 (0.000)	0.02 (0.000)
DPsi	0.16 (0.001)	0.05 (0.002)	0.24 (0.000)	0.04 (0.002)

*K* denotes the number of outcome classes. The numbers are averages over 100 training and test sets

The number of genes selected by each regularized model and the corresponding misclassification rate based on the 10 fold cross-validations repeated 100 times are provided in Table 3. Of all the methods, Enet followed by Lasso and DPsi selected the largest number of predictors, for example for EMTAB2805 that had $K=3$ outcome classes, Enet selected 636 predictors, Lasso 132 and DPsi selected 184 predictors. In contrast, DPan selected only 44 predictors as outcome associated, followed by AdaLS that selected 47 for EMTAB2805. Both these models yielded the sparsest solutions for all four datasets, but AdaLS resulted in the highest misclassification rate for two out of the four datasets. The misclassification rates of Lasso, Enet and DPan were very similar and the lowest of all methods, except for EMTAB2805 where AdaLS had a misclassification rate close to zero.

In summary, DPan identified more parsimonious models than all other methods, while maintaining excellent accuracy, similar to that of Lasso and Enet, both well established regularized regression approaches.

## Discussion

We proposed the DP-lasso, a penalized likelihood method for categorical outcomes with novel penalty weights for variable selection, particularly suited to high dimensional predictor settings. All DP weights are functions of the intra- and inter-outcome-category distances of each predictor. When observations for a particular predictor have low within outcome group variability and large variability between outcome groups, that predictor will be penalized less as it contains important information about the outcome. The DPan weights are functions of the F-statistic that is used to determine whether group means are equal in a one-way ANOVA, and the DPdb and DPsi weights use the Davies-Bouldin index and the silhouette index, respectively, two indices developed to assess the quality of clustering results. As our novel DP weights are constructed using marginal statistics, they are particularly well suited to the $Kp>N$ setting, where *K* denotes the number of outcome categories, *N* is the available sample size and *p* is the number of predictors.

In simulations with correlated predictors, DPan yielded high TPRs and low FPRs and resulted in a considerably lower number of selected predictors than all other methods we studied, including AdaLS, which also uses weights based on the marginal features of the predictors. However, the TPRs of AdaLS were considerably lower than those of the DP-lasso approaches. In low dimensions, DPan resulted in the highest TPRs, and with increasing numbers of simulated predictors and increasing correlations between predictors, the FPRs decreased. For high-dimensional simulated data, DPan had the highest TPRs with exception to the highest correlation setting, where Enet performed better in some cases. However, for very large *p* and very highly correlated predictors DPan had the lowest FPRs of all the methods. The same pattern was seen when we analyzed several single-cell RNA-sequencing datasets, all with $Kp>>N$. DPan had low misclassification rates and selected small numbers of predictors as outcome associated. While AdaLS selected similar numbers of predictors as DPan, it had higher misclassification rates.

The demonstrated sparseness of models selected using DPan is particularly desirable for molecular discovery analyses, e.g. the analysis of gene expression data, where the number of candidate genes is very large, and follow-up studies are costly.

Our comparisons do have some limitations worth mentioning. We could not include some other penalized approaches proposed in the literature for polytomous logistic regression models (Nibbering and Hastie 2022; Krishnapuram et al. 2005) as there were no readily available *R* programs. To the best of our knowledge there is no proposal for determining the optimal weights for the adaptive lasso as implemented in the *R* package *glmnet* for multinomial regression. We therefore selected the average of the coefficient estimates of the corresponding variable obtained from least squares or Ridge regression fits as weights, which may not result in the optimal performance of the adaptive lasso. We also were not able to present results for polytomous logistic regression for the relaxed lasso (Meinshausen 2007), as the algorithm did not converge in most simulations.

Other approaches for ultra-high-dimensional settings for variable screening include marginal screening, e.g. by two sample t-tests, and marginal correlation based ranking methods (Li et al. 2012; Fan and Lv 2008), followed by penalized likelihood based procedures applied to the thus pre-selected variables. A detailed numerical comparison with these approaches might be part of future work. In future work, one could also include a hyper-parameter $\gamma$ into the DP weights in (23) and use $(w^{DP})^\gamma$, similar to the adaptive lasso in (8). Another line of future work is to investigate accommodating correlations between predictors and adjusting the penalization accordingly, as was done in Tutz and Ulbricht (2009). However, the increased computational complexity of the weight calculation when pairwise or higher order correlations among predictors are accommodated needs to be balanced with the possible gain in selection performance. This is a particularly important consideration for ultra high dimensional settings.

In summary, the DP-lasso with ANOVA based weights is easy to implement and performed extremely well for variable selection in very high-dimensional settings with correlated predictors.

## Supplementary Information

Below is the link to the electronic supplementary material.

Supplementary file 1 (pdf 386 KB)

## Data Availability

The dataset EMTAB2805 (Buettner et al. 2015) is publicly available on ArrayExpress (https://www.ebi.ac.uk/biostudies/arrayexpress). GSE45719 (Deng et al. 2014), GSE48968 (Shalek et al. 2014), and GSE74596 (Engel et al. 2016) are all part of the Gene Expression Omnibus (https://www.ncbi.nlm.nih.gov/geo/) provided by the National Center for Biotechnology Information of the National Institutes of Health (NIH). Links to each publicly accessible dataset are provided in the Supplemental Material.
