# Supplementary material for: A powerful penalized multinomial logistic regression approach
Source: Comput Stat. 2025 May 25;40(8):4565–87. doi: 10.1007/s00180-025-01635-0 (PMC12552268; doi:10.1007/s00180-025-01635-0)
Supplement: Supplementary file 1 — (pdf 386 KB) [file 180_2025_1635_MOESM1_ESM.pdf]

Supplemental Material: A powerful penalized multinomial logistic regression approach

Cornelia Fuetterer[1*], Malte Nalenz[2], Thomas Augustin[2†], Ruth M. Pfeiffer[3*†]

[1*]Institute of AI and Informatics in Medicine, Technical University of Munich (TUM), TUM School of Medicine, Munich, Germany, Ismaninger Straße 22, Munich, 81675, Bavaria, Germany.
[2]Department of Statistics, Ludwig-Maximilians-University, Munich, Ludwigstraße 33, Munich, 80539, Bavaria, Germany.
[3*]Biostatistics Branch, National Cancer Institute, 9609 Medical Center Drive, Bethesda, 20892, Maryland, USA.

*Corresponding author(s). E-mail(s): cornelia.fuetterer@tum.de; pfeiffer@mail.nih.gov;
Contributing authors: malte.nlz@gmail.com; thomas.augustin@stat.uni-muenchen.de;
†These authors contributed equally to this work.

Table 1: True positive rate (TPR) and false positive rate (FPR) for $K = 7$ classes, $N = 1000$, $p = 100$ and $p^* = 10$, and $\sigma^2 = 0.5$, averaged over 100 runs. $\kappa \in \{0.5, 0.2\}$ for $j = 1, \ldots, 10$ for *structure 1*, and $j = 11, \cdots, 20$ for class 3 for *structure 2*. All remaining parameters are not associated with the outcome and are set to 0.

	TPR	FPR	TPR	FPR	TPR	FPR	TPR	FPR
	$\rho = 0$		$\rho = 0.3$		$\rho = 0.6$		$\rho = 0.9$	
$\kappa = 0.5$, *structure 1*								
Lasso	1.000	0.582	1.000	0.544	1.000	0.451	1.000	0.358
Enet	1.000	0.617	1.000	0.596	1.000	0.579	0.999	0.508
AdaRI	0.943	0.307	0.944	0.265	0.883	0.220	0.728	0.165
AdaLS	0.941	0.341	0.947	0.223	0.915	0.164	0.807	0.105
DPan	1.000	0.254	1.000	0.099	1.000	0.104	0.999	0.105
DPdb	0.932	0.339	0.895	0.278	0.847	0.221	0.679	0.164
DPsi	1.000	0.471	1.000	0.371	1.000	0.323	0.996	0.256
$\kappa = 0.5$, *structure 2*								
Lasso	0.999	0.662	1.000	0.535	0.999	0.478	0.981	0.368
Enet	0.999	0.702	1.000	0.608	1.000	0.594	0.994	0.496
AdaRI	0.927	0.360	0.938	0.253	0.898	0.218	0.742	0.180
AdaLS	0.853	0.419	0.937	0.235	0.891	0.166	0.787	0.106
DPan	0.992	0.378	1.000	0.115	1.000	0.111	0.982	0.109
DPdb	0.852	0.412	0.861	0.278	0.802	0.221	0.700	0.165
DPsi	0.991	0.549	1.000	0.357	0.999	0.322	0.982	0.263
$\kappa = 0.2$, *structure 1*								
Lasso	0.761	0.228	0.993	0.329	0.991	0.296	0.921	0.230
Enet	0.769	0.268	0.994	0.349	0.994	0.344	0.963	0.321
AdaRI	0.698	0.151	0.846	0.148	0.788	0.140	0.596	0.099
AdaLS	0.509	0.155	0.881	0.145	0.810	0.110	0.703	0.087
DPan	0.895	0.236	0.990	0.069	0.986	0.087	0.917	0.086
DPdb	0.545	0.142	0.785	0.150	0.740	0.137	0.548	0.099
DPsi	0.835	0.248	0.993	0.219	0.990	0.199	0.923	0.158
$\kappa = 0.2$, *structure 2*								
Lasso	0.246	0.106	0.976	0.331	0.963	0.307	0.911	0.252
Enet	0.362	0.180	0.978	0.377	0.978	0.366	0.959	0.342
AdaRI	0.274	0.099	0.793	0.172	0.719	0.138	0.537	0.110
AdaLS	0.219	0.101	0.836	0.162	0.780	0.122	0.684	0.095
DPan	0.555	0.222	0.962	0.104	0.954	0.106	0.908	0.097
DPdb	0.246	0.112	0.759	0.172	0.685	0.140	0.521	0.107
DPsi	0.403	0.197	0.981	0.233	0.969	0.202	0.920	0.173

Table 2: Selected number of predictors of simulated $p = 100$ for $K = 7$ classes, $N = 1000$, and $p^* = 10$, and $\sigma^2 = 0.5$, averaged over 100 runs. $\kappa = 0.5$, for $j = 1, \ldots, 10$ for *structure 1*. All remaining parameters are not associated with the outcome and are set to 0.

	$p = 100$			
	$\rho = 0$	$\rho = 0.3$	$\rho = 0.6$	$\rho = 0.9$
Lasso	69	64	55	46
Enet	72	70	68	61
adaRI	40	36	31	23
adaLS	44	31	25	18
DPan	36	19	19	19
DPdb	44	36	30	23
DPsi	57	47	42	35

Table 3: True positive rate (TPR) and false positive rate (FPR) for $K = 7$ classes, $N = 1000$, $p = 1000$, and $\sigma^2 = 0.5$, averaged over 100 runs.$\kappa \in \{0.5, 0.2\}$ for $j = 1, \ldots, 10$ for *structure 1*, and $j = 11, \cdots, 20$ for class 3 for *structure 2*. All remaining parameters are not associated with the outcome and are set to 0.

	TPR	FPR	TPR	FPR	TPR	FPR	TPR	FPR
	$\rho = 0$		$\rho = 0.3$		$\rho = 0.6$		$\rho = 0.9$	
$\kappa = 0.5$, *structure 1*								
Lasso	0.997	0.103	1.000	0.099	1.000	0.093	0.987	0.080
Enet	0.997	0.115	1.000	0.117	1.000	0.134	0.990	0.129
AdaRI	0.891	0.092	0.872	0.072	0.778	0.053	0.671	0.034
AdaLS	0.842	0.102	0.915	0.059	0.851	0.040	0.776	0.020
DPan	1.000	0.181	1.000	0.049	1.000	0.010	0.996	0.010
DPdb	0.795	0.104	0.839	0.083	0.740	0.062	0.557	0.041
DPsi	0.999	0.167	1.000	0.109	1.000	0.082	0.984	0.063
$\kappa = 0.5$, *structure 2*								
Lasso	0.730	0.084	0.997	0.113	0.995	0.095	0.968	0.078
Enet	0.813	0.127	0.996	0.133	0.995	0.120	0.987	0.134
AdaRI	0.813	0.085	0.927	0.061	0.886	0.049	0.747	0.035
AdaLS	0.555	0.094	0.870	0.063	0.833	0.036	0.736	0.020
DPan	0.972	0.206	1.000	0.077	0.999	0.010	0.988	0.010
DPdb	0.591	0.099	0.784	0.082	0.700	0.058	0.520	0.040
DPsi	0.914	0.173	0.999	0.118	0.994	0.084	0.967	0.063
$\kappa = 0.2$, *structure 1*								
Lasso	0.289	0.020	0.993	0.055	0.972	0.050	0.888	0.045
Enet	0.370	0.041	0.991	0.068	0.972	0.055	0.921	0.058
AdaRI	0.402	0.033	0.799	0.037	0.710	0.029	0.614	0.019
AdaLS	0.257	0.055	0.853	0.048	0.811	0.029	0.669	0.018
DPan	0.895	0.190	1.000	0.100	0.980	0.008	0.915	0.008
DPdb	0.339	0.042	0.741	0.055	0.641	0.038	0.432	0.026
DPsi	0.671	0.098	0.996	0.079	0.985	0.055	0.906	0.039
$\kappa = 0.2$, *structure 2*								
Lasso	0.050	0.010	0.935	0.062	0.930	0.051	0.823	0.046
Enet	0.112	0.026	0.946	0.074	0.945	0.063	0.886	0.063
AdaRI	0.110	0.019	0.763	0.038	0.717	0.026	0.613	0.020
AdaLS	0.107	0.046	0.805	0.051	0.741	0.029	0.613	0.018
DPan	0.535	0.195	0.992	0.098	0.955	0.011	0.876	0.009
DPdb	0.105	0.031	0.671	0.057	0.601	0.037	0.443	0.028
DPsi	0.266	0.080	0.966	0.090	0.957	0.054	0.867	0.041

Table 4: Selected number of predictors of simulated $p = 1000$ for $K = 7$ classes, $N = 1000$, and $p^* = 10$, and $\sigma^2 = 0.5$, averaged over 100 runs. $\kappa = 0.5$, for $j = 1, \ldots, 10$ for *structure 1*. All remaining parameters are not associated with the outcome and are set to 0.

	$p = 1000$			
	$\rho = 0$	$\rho = 0.3$	$\rho = 0.6$	$\rho = 0.9$
Lasso	113	109	103	90
Enet	125	127	144	139
adaRI	101	81	61	41
adaLS	111	68	49	28
DPan	191	59	20	19
DPdb	112	91	70	46
DPsi	177	119	92	73

Table 5: True positive rate (TPR) and false positive rate (FPR) for $K = 7$ classes, $N = 1000$, $p = 10000$ and $p^* = 10$, and $\sigma^2 = 0.5$, averaged over 100 runs. $\kappa \in \{0.5, 0.2\}$ for $j = 1, \ldots, 10$ for *structure 1*, and $j = 11, \cdots, 20$ for class 3 for *structure 2*. All remaining parameters are not associated with the outcome and are set to 0.

	TPR	FPR	TPR	FPR	TPR	FPR	TPR	FPR
	$\rho = 0$		$\rho = 0.3$		$\rho = 0.6$		$\rho = 0.9$	
$\kappa = 0.5$, *structure 1*								
Lasso	0.927	0.010	1.000	0.008	1.000	0.008	0.973	0.007
Enet	0.940	0.015	1.000	0.026	1.000	0.014	0.987	0.011
AdaRI	0.810	0.022	0.810	0.021	0.689	0.010	0.568	0.008
AdaLS	0.636	0.018	0.871	0.013	0.799	0.005	0.716	0.003
DPan	1.000	0.085	1.000	0.026	1.000	0.001	0.997	0.001
DPdb	0.716	0.040	0.748	0.030	0.663	0.013	0.452	0.008
DPsi	0.996	0.070	1.000	0.039	1.000	0.014	0.969	0.008
$\kappa = 0.5$, *structure 2*								
Lasso	0.208	0.004	0.986	0.012	0.987	0.010	0.944	0.003
Enet	0.283	0.011	0.994	0.030	0.999	0.019	0.971	0.005
AdaRI	0.684	0.019	0.899	0.016	0.812	0.012	0.612	0.003
AdaLS	0.252	0.016	0.796	0.014	0.768	0.007	0.664	0.002
DPan	0.934	0.090	1.000	0.032	0.999	0.001	0.995	0.001
DPdb	0.439	0.037	0.695	0.031	0.597	0.017	0.438	0.003
DPsi	0.799	0.070	0.994	0.042	0.990	0.018	0.926	0.004
$\kappa = 0.2$, *structure 1*								
Lasso	0.083	0.001	0.968	0.004	0.975	0.004	0.866	0.004
Enet	0.100	0.003	0.981	0.009	0.986	0.006	0.932	0.005
AdaRI	0.255	0.010	0.747	0.011	0.652	0.006	0.574	0.004
AdaLS	0.121	0.015	0.812	0.014	0.768	0.006	0.624	0.004
DPan	0.772	0.092	1.000	0.070	1.000	0.012	0.939	0.000
DPdb	0.293	0.029	0.694	0.029	0.615	0.010	0.370	0.006
DPsi	0.583	0.058	0.997	0.049	0.984	0.015	0.896	0.007
$\kappa = 0.2$, *structure 2*								
Lasso	0.003	0.001	0.854	0.005	0.894	0.004	0.790	0.004
Enet	0.019	0.002	0.886	0.010	0.962	0.007	0.908	0.006
AdaRI	0.068	0.009	0.775	0.012	0.736	0.005	0.557	0.004
AdaLS	0.064	0.015	0.721	0.015	0.740	0.007	0.602	0.004
DPan	0.377	0.094	1.000	0.071	1.000	0.013	0.871	0.001
DPdb	0.125	0.027	0.643	0.028	0.574	0.011	0.348	0.006
DPsi	0.243	0.060	0.960	0.049	0.963	0.016	0.835	0.009

Table 6: Selected number of predictors of simulated $p = 10000$ for $K = 7$ classes, $N = 1000$, and $p^* = 10$, and $\sigma^2 = 0.5$, averaged over 100 runs. $\kappa = 0.5$, for $j = 1, \ldots, 10$ for *structure 1*. All remaining parameters are not associated with the outcome and are set to 0.

	$p = 10000$			
	$\rho = 0$	$\rho = 0.3$	$\rho = 0.6$	$\rho = 0.9$
Lasso	114	146	140	128
Enet	162	269	225	192
adaRI	218	228	168	130
adaLS	188	136	86	49
DPan	864	268	20	19
DPdb	407	305	216	132
DPsi	707	402	233	159

Table 7: True positive rate (TPR) and false positive rate (FPR) for $K = 4$ classes, $N = 1000$, $p = 100$ and $p^* = 10$, and $\sigma^2 = 0.5$, averaged over 100 runs. $\kappa \in \{0.5, 0.2\}$ for $j = 1, \ldots, 10$ for *structure 1*, and $j = 11, \cdots, 20$ for class 3 for *structure 2*. All remaining parameters are not associated with the outcome and are set to 0.

	TPR	FPR	TPR	FPR	TPR	FPR	TPR	FPR
	$\rho = 0$		$\rho = 0.3$		$\rho = 0.6$		$\rho = 0.9$	
$\kappa = 0.5$, *structure 1*								
Lasso	1.000	0.440	1.000	0.403	1.000	0.334	0.977	0.244
Enet	1.000	0.458	1.000	0.481	1.000	0.423	0.991	0.386
AdaRI	0.985	0.268	0.984	0.233	0.939	0.187	0.787	0.149
AdaLS	0.988	0.245	1.000	0.146	1.000	0.108	0.980	0.088
DPan	1.000	0.175	1.000	0.096	1.000	0.092	0.979	0.088
DPdb	0.961	0.286	0.954	0.246	0.909	0.195	0.796	0.130
DPsi	1.000	0.345	1.000	0.318	0.978	0.328	0.602	0.312
$\kappa = 0.5$, *structure 2*								
Lasso	0.999	0.570	1.000	0.424	0.996	0.347	0.943	0.255
Enet	1.000	0.592	1.000	0.510	0.998	0.482	0.976	0.394
AdaRI	0.991	0.323	0.992	0.232	0.967	0.218	0.751	0.151
AdaLS	0.933	0.422	1.000	0.163	0.998	0.125	0.944	0.102
DPan	0.996	0.302	1.000	0.130	0.997	0.113	0.946	0.102
DPdb	0.917	0.437	0.896	0.251	0.856	0.185	0.738	0.132
DPsi	0.999	0.443	1.000	0.312	0.988	0.320	0.619	0.307
$\kappa = 0.2$, *structure 1*								
Lasso	0.912	0.212	0.993	0.229	0.987	0.205	0.870	0.167
Enet	0.928	0.256	0.992	0.252	0.988	0.257	0.932	0.257
AdaRI	0.866	0.200	0.883	0.140	0.827	0.125	0.572	0.083
AdaLS	0.809	0.201	0.993	0.103	0.987	0.083	0.872	0.074
DPan	0.938	0.194	0.990	0.069	0.981	0.073	0.868	0.072
DPdb	0.789	0.192	0.857	0.143	0.804	0.115	0.676	0.086
DPsi	0.934	0.226	0.997	0.175	0.992	0.165	0.841	0.174
$\kappa = 0.2$, *structure 2*								
Lasso	0.465	0.171	0.958	0.263	0.923	0.222	0.796	0.174
Enet	0.535	0.224	0.963	0.265	0.951	0.270	0.879	0.245
AdaRI	0.580	0.185	0.863	0.170	0.787	0.140	0.537	0.091
AdaLS	0.480	0.171	0.972	0.139	0.937	0.106	0.791	0.084
DPan	0.665	0.230	0.944	0.108	0.919	0.099	0.778	0.082
DPdb	0.450	0.170	0.790	0.158	0.750	0.127	0.578	0.094
DPsi	0.611	0.229	0.976	0.192	0.943	0.182	0.789	0.180

Table 8: True positive rate (TPR) and false positive rate (FPR) for $K = 4$ classes, $N = 1000$, $p = 1000$ and $p^* = 10$, and $\sigma^2 = 0.5$, averaged over 100 runs. $\kappa \in \{0.5, 0.2\}$ for $j = 1, \ldots, 10$ for *structure 1*, and $j = 11, \cdots, 20$ for class 3 for *structure 2*. All remaining parameters are not associated with the outcome and are set to 0.

	TPR	FPR	TPR	FPR	TPR	FPR	TPR	FPR
	$\rho = 0$		$\rho = 0.3$		$\rho = 0.6$		$\rho = 0.9$	
$\kappa = 0.5$, *structure 1*								
Lasso	1.000	0.070	1.000	0.072	1.000	0.062	0.963	0.049
Enet	1.000	0.073	1.000	0.083	1.000	0.077	0.990	0.091
AdaRI	0.938	0.102	0.960	0.066	0.948	0.040	0.944	0.018
AdaLS	0.975	0.111	1.000	0.047	1.000	0.024	0.971	0.008
DPan	1.000	0.145	1.000	0.009	1.000	0.009	0.972	0.008
DPdb	0.942	0.124	0.922	0.081	0.818	0.048	0.681	0.027
DPsi	1.000	0.128	1.000	0.094	0.924	0.087	0.390	0.088
$\kappa = 0.5$, *structure 2*								
Lasso	0.947	0.094	0.998	0.077	0.990	0.064	0.896	0.054
Enet	0.957	0.112	0.998	0.088	0.994	0.088	0.965	0.099
AdaRI	0.983	0.109	0.992	0.064	0.986	0.039	0.928	0.019
AdaLS	0.832	0.140	0.999	0.050	0.997	0.024	0.932	0.010
DPan	0.995	0.161	1.000	0.011	0.994	0.010	0.935	0.009
DPdb	0.768	0.143	0.846	0.079	0.750	0.048	0.630	0.029
DPsi	0.983	0.152	1.000	0.097	0.953	0.084	0.456	0.088
$\kappa = 0.2$, *structure 1*								
Lasso	0.595	0.028	0.982	0.037	0.967	0.031	0.817	0.027
Enet	0.640	0.041	0.983	0.038	0.973	0.036	0.877	0.034
AdaRI	0.660	0.064	0.892	0.047	0.875	0.025	0.803	0.012
AdaLS	0.614	0.091	0.995	0.045	0.990	0.022	0.869	0.009
DPan	0.923	0.159	1.000	0.072	0.976	0.007	0.854	0.006
DPdb	0.659	0.094	0.827	0.056	0.745	0.034	0.530	0.020
DPsi	0.831	0.092	0.996	0.063	0.990	0.043	0.713	0.040
$\kappa = 0.2$, *structure 2*								
Lasso	0.107	0.013	0.906	0.042	0.878	0.034	0.710	0.029
Enet	0.137	0.020	0.902	0.041	0.893	0.036	0.802	0.037
AdaRI	0.357	0.060	0.913	0.052	0.869	0.024	0.733	0.015
AdaLS	0.301	0.078	0.977	0.053	0.942	0.024	0.794	0.011
DPan	0.604	0.166	0.996	0.080	0.909	0.009	0.761	0.008
DPdb	0.305	0.076	0.762	0.062	0.675	0.034	0.520	0.020
DPsi	0.404	0.081	0.963	0.062	0.927	0.045	0.662	0.043

Table 9: True positive rate (TPR) and false positive rate (FPR) for $K = 4$ classes, $N = 1000$, $p = 10000$ and $p^* = 10$, and $\sigma^2 = 0.5$, averaged over 100 runs. $\kappa \in \{0.5, 0.2\}$ for $j = 1, \ldots, 10$ for *structure 1*, and $j = 11, \cdots, 20$ for class 3 for *structure 2*. All remaining parameters are not associated with the outcome and are set to 0.

	TPR	FPR	TPR	FPR	TPR	FPR	TPR	FPR
	$\rho = 0$		$\rho = 0.3$		$\rho = 0.6$		$\rho = 0.9$	
$\kappa = 0.5$, *structure 1*								
Lasso	0.999	0.008	1.000	0.010	0.999	0.010	0.940	0.008
Enet	0.999	0.011	1.000	0.013	1.000	0.016	0.967	0.015
AdaRI	1.000	0.050	1.000	0.033	0.875	0.025	0.240	0.021
AdaLS	0.918	0.033	0.935	0.025	0.936	0.017	0.922	0.009
DPan	1.000	0.056	1.000	0.002	1.000	0.001	0.975	0.001
DPdb	0.877	0.048	0.871	0.033	0.787	0.022	0.593	0.011
DPsi	0.966	0.030	1.000	0.016	1.000	0.009	0.973	0.001
$\kappa = 0.5$, *structure 2*								
Lasso	0.623	0.010	0.988	0.012	0.968	0.011	0.853	0.008
Enet	0.647	0.012	0.990	0.020	0.986	0.020	0.939	0.017
AdaRI	0.923	0.054	0.999	0.035	0.904	0.025	0.304	0.021
AdaLS	0.933	0.034	0.991	0.024	0.971	0.016	0.908	0.008
DPan	0.967	0.061	1.000	0.003	0.996	0.001	0.943	0.001
DPdb	0.624	0.050	0.798	0.033	0.752	0.021	0.571	0.011
DPsi	0.694	0.037	0.999	0.015	0.997	0.010	0.941	0.002
$\kappa = 0.2$, *structure 1*								
Lasso	0.259	0.002	0.978	0.004	0.947	0.004	0.783	0.004
Enet	0.309	0.003	0.987	0.006	0.971	0.006	0.883	0.006
AdaRI	0.756	0.047	0.994	0.034	0.967	0.020	0.615	0.014
AdaLS	0.576	0.028	0.897	0.020	0.907	0.012	0.812	0.007
DPan	0.839	0.065	0.999	0.031	0.984	0.001	0.844	0.001
DPdb	0.593	0.049	0.805	0.038	0.723	0.023	0.540	0.011
DPsi	0.546	0.034	0.995	0.022	0.982	0.014	0.881	0.007
$\kappa = 0.2$, *structure 2*								
Lasso	0.020	0.001	0.828	0.005	0.862	0.005	0.693	0.004
Enet	0.031	0.002	0.870	0.007	0.898	0.006	0.817	0.007
AdaRI	0.316	0.046	0.941	0.034	0.924	0.021	0.607	0.014
AdaLS	0.255	0.027	0.902	0.019	0.923	0.012	0.742	0.007
DPan	0.396	0.066	0.999	0.035	0.954	0.002	0.770	0.001
DPdb	0.259	0.048	0.729	0.037	0.701	0.023	0.480	0.011
DPsi	0.201	0.032	0.957	0.024	0.960	0.014	0.850	0.007

Table 10: True positive rate (TPR) and false positive rate (FPR) for $K = 4$ classes, $N = 500$, $p = 100$ and $p^* = 10$, and $\sigma^2 = 0.5$, averaged over 100 runs. $\kappa \in \{0.5, 0.2\}$ for $j = 1, \ldots, 10$ for *structure 1*, and $j = 11, \cdots, 20$ for class 3 for *structure 2*. All remaining parameters are not associated with the outcome and are set to 0.

	TPR	FPR	TPR	FPR	TPR	FPR	TPR	FPR
	$\rho = 0$		$\rho = 0.3$		$\rho = 0.6$		$\rho = 0.9$	
$\kappa = 0.5$, *structure 1*								
Lasso	0.999	0.320	1.000	0.326	0.996	0.272	0.914	0.225
Enet	0.999	0.344	1.000	0.370	0.998	0.422	0.955	0.361
AdaRI	0.958	0.251	0.916	0.195	0.856	0.164	0.673	0.130
AdaLS	0.945	0.278	0.999	0.139	0.991	0.101	0.891	0.078
DPan	0.997	0.202	1.000	0.089	0.990	0.081	0.904	0.077
DPdb	0.921	0.264	0.901	0.190	0.817	0.148	0.683	0.101
DPsi	0.999	0.311	0.997	0.301	0.873	0.290	0.457	0.270
$\kappa = 0.5$, *structure 2*								
Lasso	0.957	0.422	0.986	0.343	0.972	0.292	0.848	0.223
Enet	0.978	0.510	0.991	0.413	0.986	0.428	0.924	0.362
AdaRI	0.905	0.306	0.908	0.216	0.874	0.185	0.633	0.137
AdaLS	0.820	0.309	0.986	0.173	0.977	0.119	0.852	0.095
DPan	0.929	0.299	0.979	0.118	0.968	0.108	0.860	0.093
DPdb	0.796	0.308	0.832	0.200	0.757	0.147	0.644	0.109
DPsi	0.960	0.404	0.990	0.321	0.909	0.303	0.471	0.253
$\kappa = 0.2$, *structure 1*								
Lasso	0.506	0.112	0.943	0.182	0.899	0.173	0.716	0.140
Enet	0.596	0.171	0.949	0.214	0.919	0.215	0.830	0.225
AdaRI	0.551	0.135	0.793	0.138	0.703	0.099	0.470	0.076
AdaLS	0.452	0.140	0.936	0.096	0.912	0.076	0.736	0.066
DPan	0.734	0.179	0.927	0.058	0.886	0.061	0.720	0.061
DPdb	0.506	0.135	0.780	0.115	0.689	0.089	0.545	0.073
DPsi	0.627	0.162	0.959	0.167	0.903	0.161	0.642	0.170
$\kappa = 0.2$, *structure 2*								
Lasso	0.171	0.069	0.839	0.215	0.832	0.200	0.648	0.155
Enet	0.218	0.093	0.857	0.252	0.864	0.229	0.801	0.230
AdaRI	0.259	0.107	0.705	0.147	0.661	0.123	0.433	0.082
AdaLS	0.256	0.109	0.866	0.135	0.848	0.100	0.647	0.074
DPan	0.430	0.185	0.813	0.094	0.799	0.086	0.642	0.071
DPdb	0.272	0.116	0.701	0.129	0.603	0.106	0.501	0.076
DPsi	0.337	0.146	0.891	0.193	0.857	0.187	0.637	0.160

Table 11: True positive rate (TPR) and false positive rate (FPR) for $K = 4$ classes, $N = 500$, $p = 1000$ and $p^* = 10$, and $\sigma^2 = 0.5$, averaged over 100 runs. $\kappa \in \{0.5, 0.2\}$ for $j = 1, \ldots, 10$ for *structure 1*, and $j = 11, \cdots, 20$ for class 3 for *structure 2*. All remaining parameters are not associated with the outcome and are set to 0.

	TPR	FPR	TPR	FPR	TPR	FPR	TPR	FPR
	$\rho = 0$		$\rho = 0.3$		$\rho = 0.6$		$\rho = 0.9$	
$\kappa = 0.5$, *structure 1*								
Lasso	0.982	0.049	0.996	0.055	0.987	0.045	0.854	0.041
Enet	0.983	0.064	1.000	0.090	0.996	0.102	0.923	0.080
AdaRI	0.870	0.087	0.889	0.061	0.864	0.038	0.795	0.022
AdaLS	0.855	0.093	0.998	0.045	0.996	0.024	0.916	0.010
DPan	0.997	0.127	1.000	0.031	0.992	0.008	0.910	0.007
DPdb	0.819	0.094	0.842	0.062	0.737	0.042	0.611	0.024
DPsi	0.991	0.107	0.985	0.096	0.732	0.075	0.273	0.071
$\kappa = 0.5$, *structure 2*								
Lasso	0.488	0.043	0.957	0.057	0.922	0.051	0.761	0.043
Enet	0.599	0.083	0.975	0.116	0.967	0.097	0.884	0.082
AdaRI	0.750	0.088	0.942	0.054	0.915	0.034	0.798	0.022
AdaLS	0.571	0.094	0.984	0.045	0.978	0.025	0.860	0.011
DPan	0.849	0.145	0.997	0.038	0.978	0.010	0.862	0.009
DPdb	0.522	0.093	0.788	0.065	0.720	0.041	0.565	0.024
DPsi	0.742	0.112	0.977	0.094	0.809	0.077	0.287	0.071
$\kappa = 0.2$, *structure 1*								
Lasso	0.205	0.012	0.872	0.028	0.858	0.030	0.650	0.025
Enet	0.227	0.024	0.902	0.036	0.903	0.039	0.813	0.040
AdaRI	0.372	0.051	0.776	0.041	0.747	0.024	0.606	0.015
AdaLS	0.313	0.068	0.913	0.045	0.926	0.023	0.735	0.010
DPan	0.667	0.139	0.980	0.069	0.923	0.015	0.710	0.005
DPdb	0.389	0.076	0.744	0.053	0.653	0.028	0.506	0.019
DPsi	0.435	0.062	0.904	0.055	0.847	0.046	0.433	0.040
$\kappa = 0.2$, *structure 2*								
Lasso	0.028	0.009	0.717	0.030	0.730	0.029	0.570	0.027
Enet	0.043	0.016	0.775	0.044	0.810	0.041	0.745	0.045
AdaRI	0.145	0.044	0.762	0.040	0.745	0.028	0.577	0.016
AdaLS	0.166	0.067	0.847	0.051	0.845	0.021	0.675	0.011
DPan	0.348	0.144	0.972	0.074	0.850	0.014	0.642	0.006
DPdb	0.155	0.070	0.654	0.055	0.642	0.031	0.449	0.018
DPsi	0.132	0.052	0.844	0.057	0.811	0.049	0.458	0.042

Table 12: True positive rate (TPR) and false positive rate (FPR) for $K = 4$ classes, $N = 500$, $p = 10000$ and $p^* = 10$, and $\sigma^2 = 0.5$, averaged over 100 runs. $\kappa \in \{0.5, 0.2\}$ for $j = 1, \ldots, 10$ for *structure 1*, and $j = 11, \cdots, 20$ for class 3 for *structure 2*. All remaining parameters are not associated with the outcome and are set to 0.

	TPR	FPR	TPR	FPR	TPR	FPR	TPR	FPR
	$\rho = 0$		$\rho = 0.3$		$\rho = 0.6$		$\rho = 0.9$	
$\kappa = 0.5$, *structure 1*								
Lasso	0.837	0.006	0.992	0.007	0.974	0.008	0.818	0.007
Enet	0.853	0.008	0.997	0.018	0.985	0.015	0.919	0.014
AdaRI	0.763	0.023	0.840	0.019	0.806	0.015	0.739	0.009
AdaLS	0.723	0.020	0.994	0.011	0.994	0.007	0.942	0.003
DPan	0.976	0.035	0.999	0.011	0.996	0.001	0.935	0.001
DPdb	0.711	0.031	0.822	0.023	0.731	0.017	0.548	0.009
DPsi	0.937	0.031	0.960	0.024	0.610	0.019	0.175	0.014
$\kappa = 0.5$, *structure 2*								
Lasso	0.156	0.004	0.900	0.009	0.852	0.008	0.677	0.007
Enet	0.212	0.011	0.947	0.027	0.924	0.021	0.839	0.014
AdaRI	0.567	0.022	0.927	0.018	0.881	0.015	0.738	0.010
AdaLS	0.364	0.021	0.974	0.013	0.959	0.007	0.881	0.004
DPan	0.644	0.038	0.997	0.013	0.964	0.001	0.869	0.001
DPdb	0.389	0.032	0.744	0.023	0.638	0.016	0.536	0.010
DPsi	0.531	0.030	0.961	0.026	0.704	0.020	0.192	0.015
$\kappa = 0.2$, *structure 1*								
Lasso	0.045	0.001	0.802	0.003	0.819	0.004	0.635	0.004
Enet	0.084	0.004	0.848	0.006	0.908	0.006	0.782	0.006
AdaRI	0.250	0.020	0.765	0.016	0.794	0.012	0.632	0.008
AdaLS	0.165	0.021	0.907	0.015	0.933	0.011	0.776	0.007
DPan	0.411	0.040	0.983	0.030	0.998	0.012	0.752	0.001
DPdb	0.266	0.032	0.688	0.027	0.669	0.019	0.498	0.011
DPsi	0.280	0.027	0.914	0.023	0.827	0.018	0.359	0.012
$\kappa = 0.2$, *structure 2*								
Lasso	0.002	0.001	0.581	0.004	0.664	0.005	0.503	0.004
Enet	0.012	0.002	0.649	0.008	0.784	0.007	0.714	0.006
AdaRI	0.083	0.019	0.749	0.016	0.755	0.011	0.559	0.007
AdaLS	0.074	0.021	0.780	0.017	0.867	0.011	0.680	0.006
DPan	0.155	0.040	0.945	0.030	0.984	0.012	0.692	0.001
DPdb	0.110	0.033	0.626	0.026	0.588	0.019	0.416	0.010
DPsi	0.111	0.027	0.789	0.024	0.782	0.018	0.355	0.012

Table 13: True positive rate (TPR) and false positive rate (FPR) for $K = 2$ classes, $N = 500$, $p = 100$ and $p^* = 10$, and $\sigma^2 = 0.5$, averaged over 100 runs (up to 187 runs were needed to obtain 100 for which MCP converged). $\kappa \in \{0.5, 0.2\}$ for $j = 1, \ldots, 10$ and all remaining parameters are not associated with the outcome and are set to 0.

	TPR	FPR	TPR	FPR	TPR	FPR	TPR	FPR
	$\rho = 0$		$\rho = 0.3$		$\rho = 0.6$		$\rho = 0.9$	
$\kappa = 0.5$								
MCP	0.93	0.04	0.89	0.02	0.73	0.02	0.13	0.01
SCAD	0.97	0.08	0.96	0.05	0.88	0.03	0.48	0.02
Lasso	0.98	0.11	0.98	0.08	0.92	0.06	0.63	0.04
Enet	0.98	0.12	0.99	0.09	0.96	0.11	0.82	0.09
AdaRI	0.98	0.09	0.95	0.07	0.86	0.06	0.50	0.04
AdaLS	0.98	0.09	0.98	0.04	0.92	0.02	0.62	0.02
DPan	0.96	0.07	0.97	0.03	0.90	0.02	0.62	0.02
DPdb	0.98	0.10	0.98	0.05	0.93	0.03	0.64	0.02
DPsi	0.90	0.05	0.97	0.03	0.92	0.02	0.63	0.02
$\kappa = 0.2$								
MCP	0.22	0.01	0.53	0.02	0.34	0.01	0.11	0.01
SCAD	0.29	0.03	0.68	0.04	0.57	0.02	0.20	0.01
Lasso	0.32	0.03	0.71	0.05	0.63	0.04	0.41	0.03
Enet	0.36	0.05	0.73	0.06	0.70	0.07	0.59	0.07
AdaRI	0.53	0.08	0.66	0.06	0.55	0.05	0.33	0.04
AdaLS	0.50	0.07	0.74	0.04	0.64	0.02	0.42	0.02
DPan	0.50	0.06	0.71	0.03	0.60	0.02	0.40	0.01
DPdb	0.50	0.07	0.75	0.04	0.64	0.03	0.43	0.02
DPsi	0.41	0.05	0.71	0.03	0.62	0.02	0.41	0.01

Table 14: True positive rate (TPR) and false positive rate (FPR) for $K = 2$ classes, $N = 500$, $p = 1000$ and $p^* = 10$, and $\sigma^2 = 0.5$, averaged over 100 runs (up to 127 runs for each setting were needed to obtain 100 for which MCP converged). $\kappa \in \{0.5, 0.2\}$ for $j = 1, \ldots, 10$ and all remaining parameters are not associated with the outcome and are set to 0.

	TPR	FPR	TPR	FPR	TPR	FPR	TPR	FPR
	$\rho = 0$		$\rho = 0.3$		$\rho = 0.6$		$\rho = 0.9$	
$\kappa = 0.5$								
MCP	0.78	0.01	0.87	0.00	0.68	0.00	0.13	0.00
SCAD	0.84	0.02	0.95	0.01	0.87	0.01	0.56	0.00
Lasso	0.86	0.02	0.96	0.01	0.89	0.01	0.61	0.01
Enet	0.87	0.02	0.98	0.02	0.94	0.02	0.79	0.01
AdaRI	0.94	0.05	0.97	0.03	0.91	0.02	0.63	0.00
AdaLS	0.94	0.05	0.98	0.02	0.93	0.01	0.63	0.00
DPan	0.93	0.04	0.98	0.01	0.89	0.00	0.61	0.00
DPdb	0.92	0.05	0.98	0.02	0.93	0.01	0.64	0.00
DPsi	0.86	0.03	0.98	0.01	0.89	0.00	0.62	0.00
$\kappa = 0.2$								
MCP	0.06[1]	0.00[1]	0.45[2]	0.00[2]	0.30[3]	0.00[3]	0.08[4]	0.00[4]
SCAD	0.09[1]	0.00[1]	0.63[2]	0.01[2]	0.58[3]	0.00 [3]	0.22[4]	0.00[4]
Lasso	0.09	0.00	0.64	0.01	0.61	0.01	0.39	0.00
Enet	0.12	0.01	0.68	0.01	0.69	0.01	0.54	0.01
AdaRI	0.41	0.05	0.71	0.04	0.65	0.03	0.40	0.01
AdaLS	0.41	0.05	0.74	0.03	0.69	0.01	0.46	0.00
DPan	0.41	0.05	0.82	0.03	0.75	0.01	0.42	0.00
DPdb	0.40	0.05	0.74	0.03	0.69	0.01	0.45	0.00
DPsi	0.32	0.03	0.78	0.02	0.74	0.01	0.42	0.00

Table 15: True positive rate (TPR) and false positive rate (FPR) for $K = 2$ classes, $N = 500$, $p = 10000$ and $p^* = 10$, and $\sigma^2 = 0.5$, averaged over 100 runs (up to 107 runs for each setting were needed to obtain 100 for which MCP converged). $\kappa \in \{0.5, 0.2\}$ for $j = 1, \ldots, 10$ and all remaining parameters are not associated with the outcome and are set to 0.

	TPR	FPR	TPR	FPR	TPR	FPR	TPR	FPR
	$\rho = 0$		$\rho = 0.3$		$\rho = 0.6$		$\rho = 0.9$	
$\kappa = 0.5$								
MCP	0.455	0.001	0.846	0.001	0.656	0.000	0.656	0.000
SCAD	0.550	0.002	0.951	0.002	0.881	0.002	0.881	0.002
Lasso	0.577	0.002	0.957	0.002	0.889	0.002	0.889	0.002
Enet	0.626	0.004	0.982	0.004	0.946	0.003	0.946	0.003
AdaRI	0.815	0.011	0.972	0.008	0.899	0.005	0.899	0.005
AdaLS	0.812	0.011	0.973	0.007	0.918	0.003	0.918	0.003
DPan	0.800	0.010	0.985	0.003	0.912	0.000	0.912	0.000
DPdb	0.812	0.011	0.971	0.007	0.912	0.004	0.912	0.004
DPsi	0.743	0.009	0.983	0.003	0.924	0.000	0.924	0.000
$\kappa = 0.2$								
MCP	0.004	0.000	0.427	0.000	0.293	0.000	0.081	0.000
SCAD	0.017	0.000	0.599	0.001	0.597	0.001	0.231	0.000
Lasso	0.015	0.000	0.601	0.001	0.615	0.001	0.396	0.001
Enet	0.033	0.002	0.643	0.002	0.682	0.002	0.557	0.001
AdaRI	0.194	0.012	0.710	0.010	0.678	0.008	0.474	0.005
AdaLS	0.196	0.012	0.704	0.010	0.692	0.007	0.501	0.004
DPan	0.175	0.010	0.756	0.008	0.822	0.004	0.597	0.001
DPdb	0.197	0.012	0.707	0.010	0.693	0.007	0.483	0.004
DPsi	0.165	0.010	0.750	0.008	0.822	0.005	0.617	0.001

Table 16: Websites where datasets used in example are available for download.

EMTAB2805	https://www.ebi.ac.uk/biostudies/arrayexpress/studies/E-MTAB-2805
GSE45719	https://www.ncbi.nlm.nih.gov/geo/query/acc.cgi?acc=GSE45719
GSE48968	https://www.ncbi.nlm.nih.gov/geo/query/acc.cgi?acc=GSE48968
GSE74596	https://www.ncbi.nlm.nih.gov/geo/query/acc.cgi?acc=GSE74596

Plots for predictor-specific weights for simulation scenarios $p = 100$

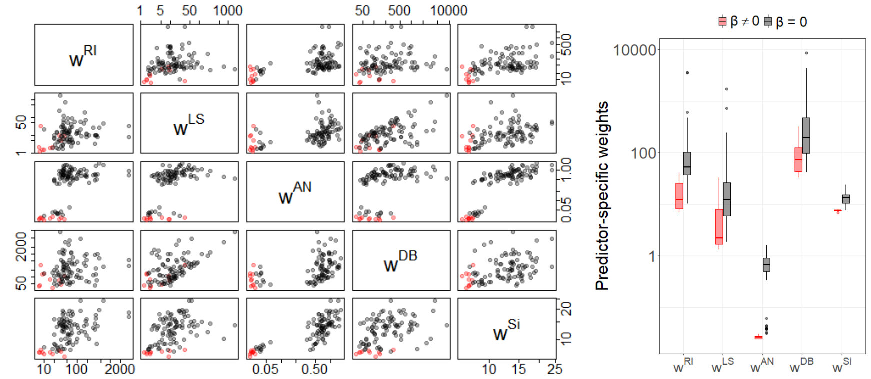

Fig. 1: Weights for data generated from *scenario 1* with $\rho = 0.3$, $\kappa = 0.5$, $p = 100$, $N = 1000$ for outcomes with $K = 7$ categories. Red dots/boxes correspond to variables that were simulated as outcome associated. All axes are on the logarithmic scale.

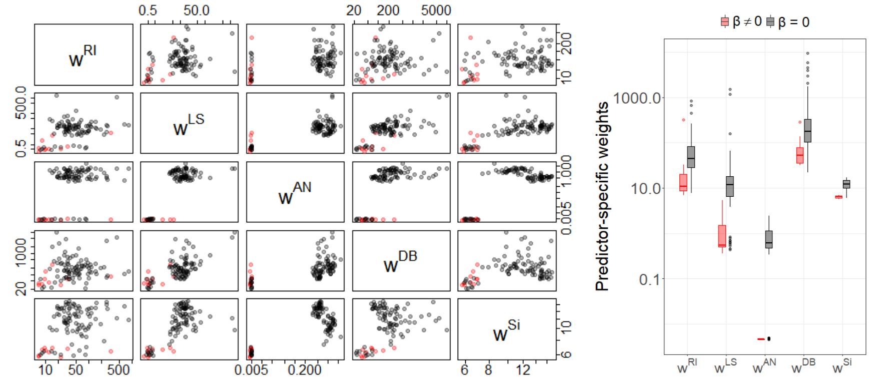

Fig. 2: Weights for data generated from *scenario 1* with $\rho = 0.9$, $\kappa = 0.5$, $p = 100$, $N = 1000$ for outcomes with $K = 7$ categories. Red dots/boxes correspond to variables that were simulated as outcome associated. All axes are on the logarithmic scale.

Plots for predictor-specific weights for simulation scenarios $p = 10000$

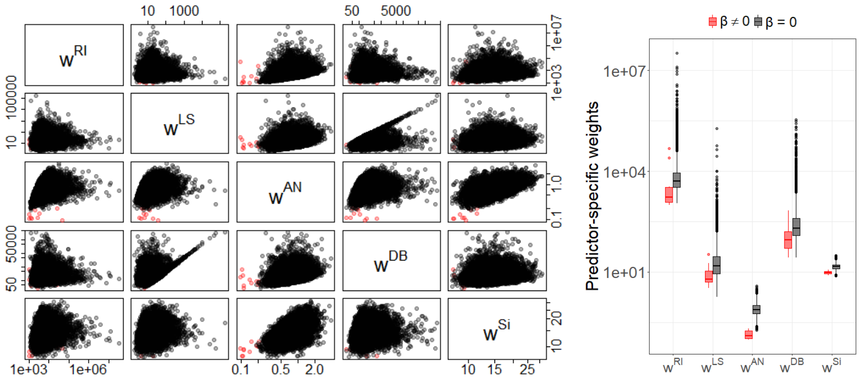

Fig. 3: Weights for data generated from *scenario 1* with $\rho = 0$, $\kappa = 0.5$, $p = 10000$, $N = 1000$ for outcomes with $K = 7$ categories. Red dots/boxes correspond to variables that were simulated as outcome associated. All axes are on the logarithmic scale.

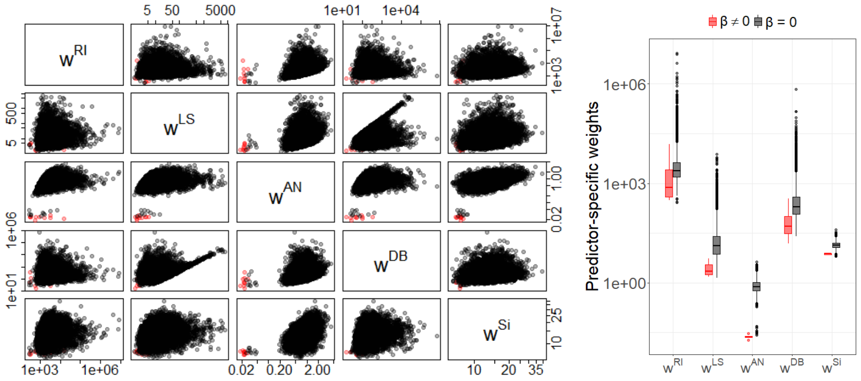

Fig. 4: Weights for data generated from *scenario 1* with $\rho = 0.3$, $\kappa = 0.5$, $p = 10000$, $N = 1000$ for outcomes with $K = 7$ categories. Red dots/boxes correspond to variables that were simulated as outcome associated. All axes are on the logarithmic scale.

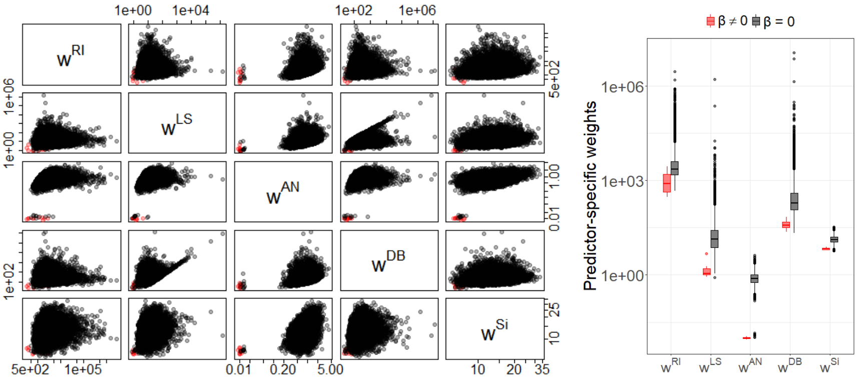

Fig. 5: Weights for data generated from *scenario 1* with $\rho = 0.6$, $\kappa = 0.5$, $p = 10000$, $N = 1000$ for outcomes with $K = 7$ categories. Red dots/boxes correspond to variables that were simulated as outcome associated. All axes are on the logarithmic scale.

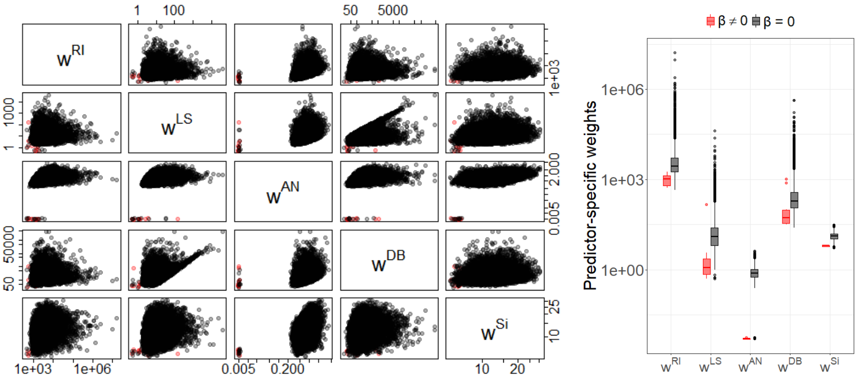

Fig. 6: Weights for data generated from *scenario 1* with $\rho = 0.9$, $\kappa = 0.5$, $p = 10000$, $N = 1000$ for outcomes with $K = 7$ categories. Red dots/boxes correspond to variables that were simulated as outcome associated. All axes are on the logarithmic scale.
